# A Topological Representation of Branching Neuronal Morphologies

**DOI:** 10.1007/s12021-017-9341-1

**Published:** 2017-10-03

**Authors:** Lida Kanari, Paweł Dłotko, Martina Scolamiero, Ran Levi, Julian Shillcock, Kathryn Hess, Henry Markram

**Affiliations:** 1 Blue Brain Project-EPFL, Geneva, Switzerland; 2 Department of Mathematics, Swansea University, Swansea, UK; 3 Laboratory for Topology and Neuroscience at the Brain Mind Institute, EPFL, Geneva, Switzerland; 4 Institute of Mathematics, University of Aberdeen, Aberdeen, UK

**Keywords:** Topological data analysis, Neuronal morphologies, Branching morphology, Clustering trees

## Abstract

Many biological systems consist of branching structures that exhibit a wide variety of shapes. Our understanding of their systematic roles is hampered from the start by the lack of a fundamental means of standardizing the description of complex branching patterns, such as those of neuronal trees. To solve this problem, we have invented the Topological Morphology Descriptor (TMD), a method for encoding the spatial structure of any tree as a “barcode”, a unique topological signature. As opposed to traditional morphometrics, the TMD couples the topology of the branches with their spatial extents by tracking their topological evolution in 3-dimensional space. We prove that neuronal trees, as well as stochastically generated trees, can be accurately categorized based on their TMD profiles. The TMD retains sufficient global and local information to create an unbiased benchmark test for their categorization and is able to quantify and characterize the structural differences between distinct morphological groups. The use of this mathematically rigorous method will advance our understanding of the anatomy and diversity of branching morphologies.

**Electronic supplementary material:**

The online version of this article (10.1007/s12021-017-9341-1) contains supplementary material, which is available to authorized users.

## Introduction

The analysis of complex branching structures, such as branched polymers (Alexandrowicz 1985), viscous fingering (Agam et al. 2002), and fractal trees (Mandelbrot and Freeman 1983), is essential for understanding a great variety of physical and biological processes. For example, the fundamental units of the nervous system, neurons, possess highly ramified arborizations (Jan and Jan 2010) that are thought to reflect their involvement in different computational tasks (Cuntz et al. 2007; Zomorrodi et al. 2010; Van Elburg and Van Ooyen 2010; Ferrante et al. 2013). In order to understand the properties of branching morphologies we must study the differences between distinct arbor types. Much effort has therefore been devoted to grouping morphologies into distinct classes (DeFelipe et al. 2013; Markram et al. 2004; The Petilla Interneuron Nomenclature Group P 2008), a categorization process that is important in many fields (Lyons et al. 1999). However, an efficient method for quantitatively analyzing the morphology of such structures has proved difficult to establish.

In general, the properties of branching morphologies have been rigorously studied in two extreme cases: in the limit of the full complexity of the structures (Carlsson 2009), where the entire set of points is used, and in the opposite limit of feature extraction (DeFelipe et al. 2013; Gomez-Gil et al. 2008; Blackman et al. 2014), where a (typically small) number of selected morphometrics (i.e., statistical features) are extracted from the morphology.

Topological data analysis (TDA) has been shown to reliably identify geometric objects based on a sampled point cloud when they are built out of well-understood pieces, such as spheres, cylinders and tori (Carlsson 2009). It suffers, however, from the deficiency that reliable grouping of complex geometric trees by standard TDA methods, such as Rips complexes (Edelsbrunner and Harer 2008), requires thousands of sampled points, which is expensive in terms of both computational complexity and memory requirements.

Feature extraction is thus the only currently feasible solution to establishing a more quantitative approach to analyzing branching morphologies (Scorcioni et al. 2008; Ling et al. 2012; The Petilla Interneuron Nomenclature Group P 2008). While this approach has been efficiently used in specific fields of image recognition (Schurer 1994), the extreme diversity of the branching patterns of neurons (Markram et al. 2004) makes it difficult to identify an optimal set of statistical features that can reliably describe all their shapes. Neuronal classification has traditionally focused on visually distinguishing the shapes observed under a microscope (Masseroli et al. 1993), a method that is subject to large variation between experts (DeFelipe et al. 2013).

**Fig. 1:**
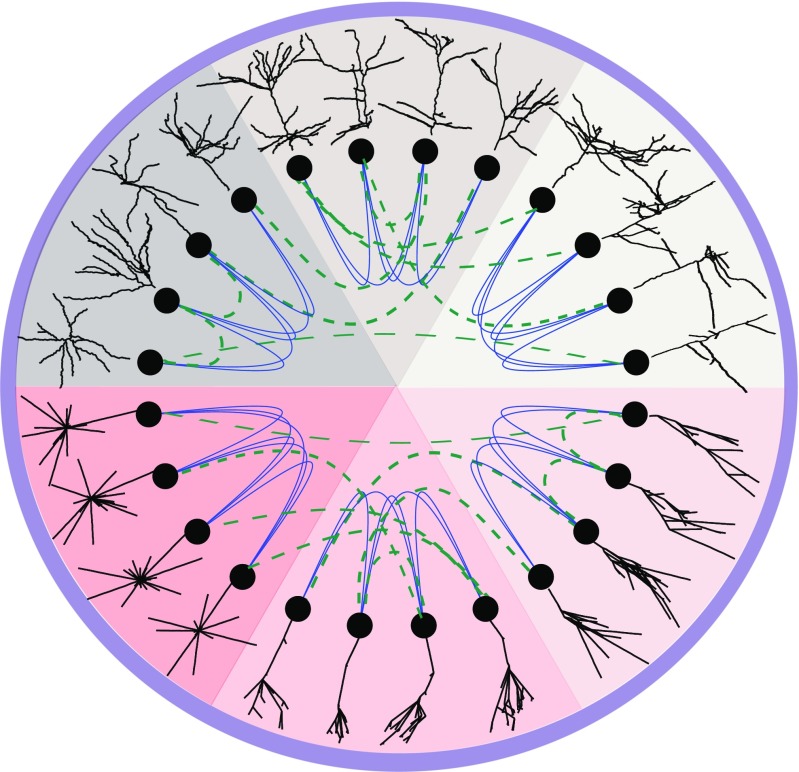
Illustration of the separation of similar tree structures into distinct groups, using topological analysis. The colored pie segments show six distinct tree types: three neuronal types (upper half) and three artificial ones (lower half). The thick blue lines show that our topological analysis can reliably separate similar-looking trees into groups. It is accurate both for artificially-generated trees and neuronal morphologies. The dashed green lines show that classification using an improper set of user-selected features (number of branches, total length) cannot distinguish the correct groups

For this reason, experts generate a digital version of a cell’s structure - a neuronal reconstruction (Dieter 2000) as a set of points in $\mathbb {R}^{3}$ sampled along each branch, together with edges connecting adjacent pairs of points. This reconstruction is a mathematical tree that represents the neuron’s morphology and can be used for the extraction of its morphological properties. To avoid overfitting, which is a result of using a large number of features when few individual cells are available, feature selection is performed by experts who identify the relevant morphometrics for each group of cells. Many sophisticated variants of the standard morphological features have been proposed over the years, such as tree asymmetry (Van Pelt et al. 1991, 2001, 2005), centrifugal ordering (Van Pelt et al. 1989) and Strahler ordering (Strahler 1952; Berry and Bradley 1976; Ledderose et al. 2014), to describe the topology of branching structures. However none of those measurements preserves the correlations between distinct features. In addition, feature selection is subjective, and alternative sets of morphometrics result in different classifications (DeFelipe et al. 2013), as illustrated in Fig. 1 (see also SI: Figs. S1-S2), since the statistical features commonly overlap even across markedly different morphological types. This is a direct consequence of the significant loss of information introduced by feature selection, as the dimensionality of the data is substantially reduced.

As a result, neither using the full point cloud of the trees nor performing expert-dependent feature selection are suitable to reliably study complex branching morphologies. In order to address this issue, we propose a standardized topological descriptor, the Topological Morphology Descriptor (TMD), of any branching morphology. The TMD algorithm encodes the branching pattern of the morphology by discarding local fluctuations with little information content, such as the position of the nodes between branch points and thus reduces the computational complexity of a tree. The TMD couples the topology of the branching structure with the embedding in the metric space, encoding the overall shape of the tree. Note that the TMD is not a complete invariant that fully describes the original tree, but a simplification that retains enough information to perform well in the proposed discrimination tasks, by mapping the tree to a topological representation with less information loss than the usual morphometrics.

The TMD algorithm takes as input the partially ordered set of branch points (nodes with more than one child) and leaves (nodes with no children) of the tree, where the order is given by the parent-child relation, and produces a multi-set of intervals on the real line known as a *persistence barcode* (Carlsson 2009), Fig. 2b. Each interval encodes the lifetime of a connected component in the underlying structure (see Glossary), identifying when a branch is first detected (birth) and when it connects to a larger subtree (death). This information can be equivalently represented in a *persistence diagram* (Carlsson 2009), Fig. 2c in which the pair of birth-death times determines a point in the real plane. Either representation greatly simplifies the mathematical analysis of the trees.

**Fig. 2:**
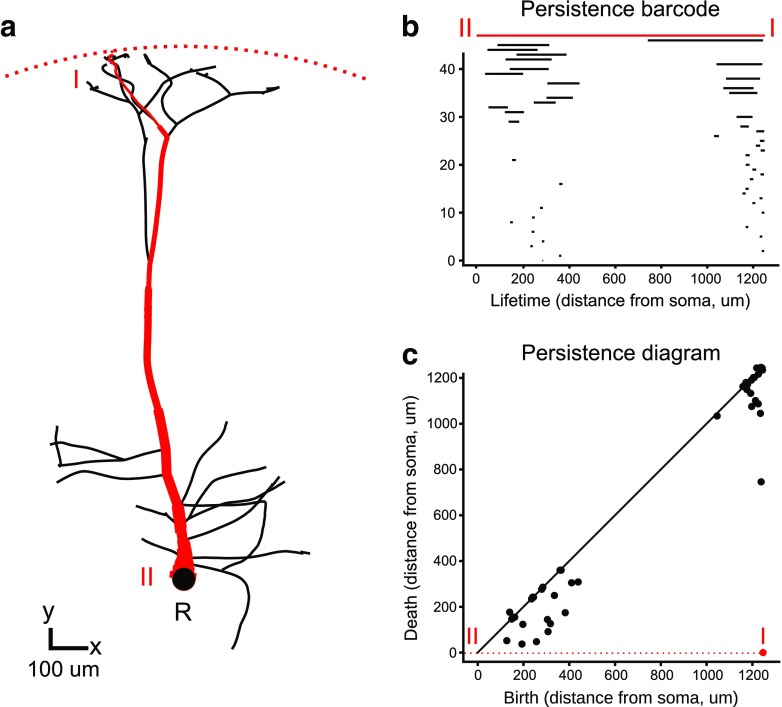
Application of topological analysis to a neuronal tree (**A**) showing the largest persistent component (red). The persistence barcode (**B**) represents each component as a horizontal line whose endpoints mark its birth and death in units that depend on the choice of the function *f* used for the ordering of the nodes of the tree. In our case, it is radial distance of the nodes from the root (R), so the units are microns. The largest component is again shown in red together with its birth (I) and death (II). This barcode can be equivalently represented as points in a persistence diagram (**C**) where the birth (I) and death (II) of a component are the X and Y coordinates of a point respectively (in red). The diagonal line is a guide to the eye and marks points with the same birth and death time

This approach provides a simplified comparison process, since distances inspired by persistent homology theory (Carlsson 2009) can be defined between the outputs of the TMD algorithm (see SI: Distances between persistence diagrams). Existing methods for computing distances between trees, such as the *edit distance* (Bille 2005), the *sequence representation* (Gillette and Ascoli 2015; Gillette et al. 2015), the *blastneuron distance* (Wan et al. 2015) and the *functional distortion distance* (Bauer et al. 2014), are in general not universally appropriate, and therefore not biologically useful, and computationally expensive (see SI:Distances between trees).

Our method, in contrast, is applicable to any tree-like structure. We demonstrate its generality by applying it first to a collection of artificial random trees, (see SI: Random trees generation), and then to various groups of neuronal trees (see Information Sharing Statement). Our results show that the TMD of tree shapes can be used effectively to assign a reliability measure to different proposed groupings of random and neuronal trees (Fig. 1). Provided that the available set of morphologies is representative of the biological diversity, we generate a diversity profile (Leinster and Cobbold 2012) that reflects the abundance of species as well as their differences, in order to further investigate the effects of different classification schemes (see SI: Diversity Index).

## Methods

The extraction of the barcode from an embedded tree *T* is described by the TMD algorithm. Let *T* be a rooted, and therefore oriented, tree (Knuth 1998), embedded in $\mathbb {R}^{3}$. Note that the operation described here is generalizable to trees embedded in any metric space. We denote by $N := B \cup L$ the set of nodes of *T*, which is the union of the set of branch points *B* and the set of leaves *L*. In the case of a neuron, the root *R* is the node representing the soma. Each node $n \in N$ has references to its parent, i.e., the first node on the path toward the root, and to its children. Nodes with the same parent are called siblings. 
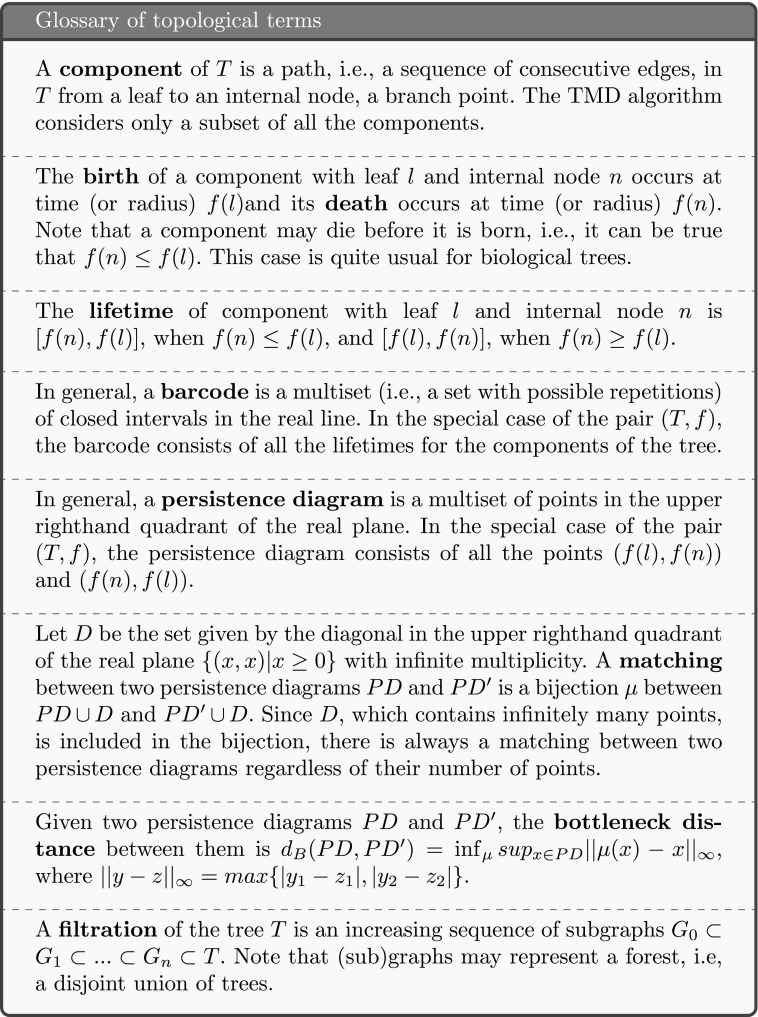


Let *f* be a real-valued function defined on the set of nodes of *T*. Any function *f* that is defined on the nodes of *T* can be used with the TMD algorithm, such as the radial distance, the path distance, the branch length, or the branch order (see SI, Fig. S4). Alternative functions should serve to reveal shape characteristics that are independent from each other and therefore be suitable for different tasks. For the purpose of this study we define *f* to be the radial distance from the root *R*. For each $n\in N$, let $T_{n}$ denote the subtree with root at the node *n*, and $L_{n}$ the set of leaves of $T_{n}$. We define a function $v \colon N \to \mathbb {R}$, computed by the TMD algorithm, by $v(n) = \max \{ f(x) \,|\, x\in L_{n} \}$. An ordering of siblings can then be defined based on *v*: if $n_{1}, n_{2} \in N$, are siblings and $v(n_{1}) < v(n_{2})$, then $n_{1}$ is younger than $n_{2}$.

The algorithm is initialized by setting the value of $v(l), l \in L$ equal to the value of $f(l)$. The leaves $l \in L$ are added to a set of nodes, denoted *A*, which keeps a record of the active nodes. Following the path of each leaf $l \in L$ toward the root *R*, all but the oldest (with respect to *v*) siblings are killed, i.e., removed from *A*, at each branch point. If siblings have the same value *v* it is equivalent to kill any one of them. For each killed component one interval (birth-death) is added to the persistence barcode (Fig. 2). The older sibling $c_{m}$ is replaced by its parent in *A* and the value $v(p)$ of its parent is set to $f(c_{m})$. This operation is applied iteratively to all the nodes until the root *R* is reached. At this point *A* contains only one component, the largest one. 
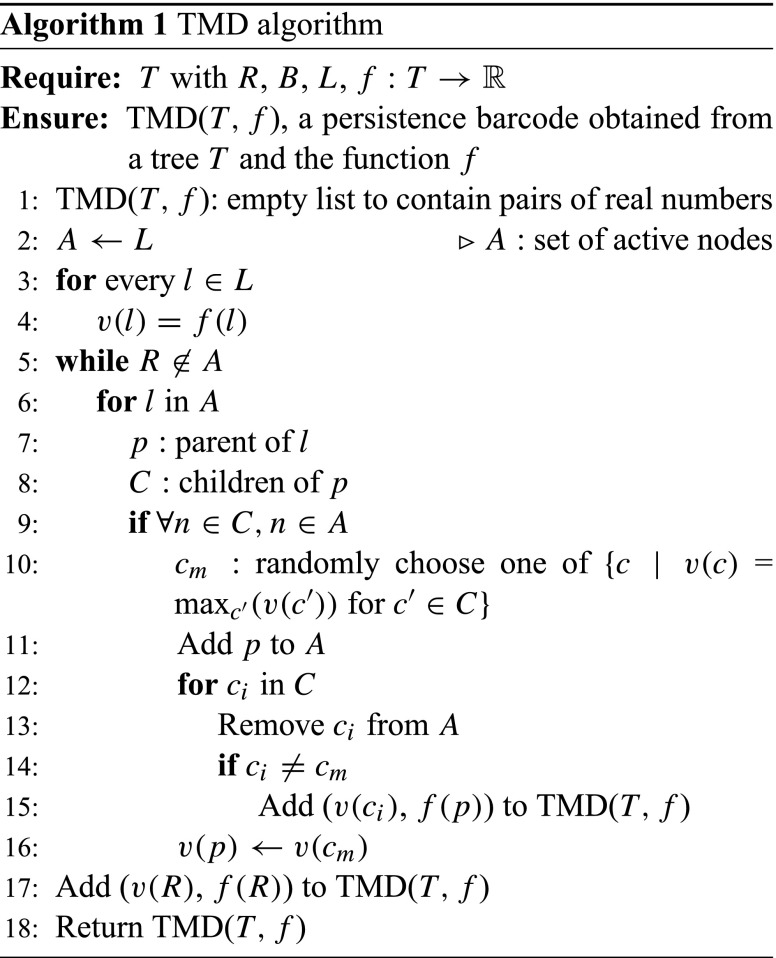


When all the branches are outgoing, i.e., the radial distance of the origin of a branch is smaller than the radial distance of its terminal point, the TMD algorithm is equivalent to computing the barcode associated to a filtration of concentric spheres of decreasing radii, centered at *R* (Fig. 2). In this case, the birth time of a component is the supremum of the radii of the spheres that do not contain the entire component. The death time is the infimum of the radii of the spheres that contain the branch point at which the component merges with a longer one.

The computational complexity of the TMD algorithm is linear in the number of nodes. Note that the *if* statement in line 9 of the algorithm is critical for the linear complexity. The number of currently active children is saved at each parent node to avoid quadratic complexity.

This process results in a set of intervals on the real line, each of which represents the lifetime of one component of the tree. The TMD algorithm that associates a persistence barcode TMD($T,f$) to a tree *T* is invariant under rotations and translations, as long as the function *f* is also. In this paper, *f* is the radial distance from *R* and as such it is invariant under rotations about the root and rigid translations of the tree in $\mathbb {R}^{3}$.

The most common topological metric that is used to compare persistence diagrams is the *bottleneck distance* (Edelsbrunner and Harer 2008), denoted $d_{B}$. Given a matching (i.e., a bijection) between two persistence diagrams $D_{1}$, $D_{2}$, we define the $L_{\infty }$ distance as the maximum distance between matched points. The bottleneck distance $d_{B}(D_{1}, D_{2})$ is the infimum over all $L_{\infty }$ distances for the possible matchings between the two persistence diagrams (Edelsbrunner and Harer 2008).

We prove that TMD: $(T, f) \mapsto $ TMD($T, f$) is stable with respect to the bottleneck distance (see SI: Stability of TMD). For $\epsilon $-small modifications of certain types in the tree *T*, the persistence diagram TMD($T, f$) is not modified more than $\mathcal {O}(\epsilon )$. In particular, the method is robust with respect to small perturbations in the positions of the nodes and the addition/ deletion of small branches.

However, none of the standard topological distances between persistence diagrams is appropriate for the comparison of neuronal trees. The bottleneck distance as well as distances stable with respect to it, such as the *persistence distortion distance* (Dey et al. 2015) (see SI: Distances between trees) cannot distinguish diagrams that differ in their short components, which are nevertheless important for the distinction of neuronal morphologies.

We therefore define in the space of the barcodes an alternative distance $d_{Bar}$ that we use to compare branching morphologies. For each barcode we generate a density profile as follows: $\forall x \in \mathbb {R}$ the value of the histogram is the number of intervals that contain *x*, i.e., the number of components alive at that point. The TMD-distance between two barcodes TMD($T_{1}, f$) and TMD($T_{2}, f$) is defined as the integral of the absolute differences between the density profiles of the barcodes. This distance is not stable for a large number of $\epsilon $-perturbations of the tree, but it is the only distance we are aware of that succeeds in capturing the differences between the short components of persistence barcodes. This distance is similar to Sholl analysis (Sholl 1953) with a few fundamental differences (see SI: Distances between neurons). However, since this density profile collapses the barcodes into a one-dimensional distribution, it fails to capture the local differences between the branching structures of similar neuronal trees.

For this reason, the persistence diagram was also converted into an *unweighted persistence image*, inspired by persistence images introduced in Adams et al. (2016). We choose to use unweighted persistence images, since points close to the diagonal, which represent short components, are important for the discrimination of the neuronal trees, and these points are ignored in the weighted persistence images. The unweighted persistence image representation allows the construction of an average image for groups of trees, which is useful for quantifying the differences between tree types, since we are not aware of any unambiguous and computationally feasible calculation of an average of persistence barcodes or diagrams. This method is based on the discretization of a sum of Gaussian kernels (Scott 2008), centered at the points of the persistence diagram. This discretization generates a matrix of pixel values, encoding the persistence diagram in a vector, called the unweighted persistence image. Machine learning tools, such as decision trees and support vector machines can then be applied to this vector for the classification of the persistence diagrams. Note that the unweighted persistence images, unlike the persistence images defined in Adams et al. (2016), do not satisfy stability for the Euclidean distance between their vectors with respect to the perturbations of trees that we consider (see SI: Stability of TMD).

## Results

We demonstrate the discriminative power of the TMD by applying it to four examples of increasing complexity. The first application is the grouping of artificial random trees that provide a well-defined test case to explore the method’s performance. The random trees are generated by a constrained stochastic algorithm (see SI: Random trees generation) and have properties that can be precisely modified. Next, we have analyzed datasets of more biological relevance: neurons from different species, downloaded from Ascoli et al. (2007), and distinct types of trees obtained from several morphological types of rat cortical pyramidal cells (Romand et al. 2011) (see Information Sharing Statement). This last example is interesting because, although there is biological support for their separation into distinct groups, no rigorous mathematical model has been proposed for their objective classification. Finally, we used the TMD-distance to rank automatic reconstructions from the BigNeuron project (Peng et al. 2015). We thereby illustrate the usefulness of the TMD across non-trivial examples.

Mathematical random trees are defined by a set of parameters that constrain their shape: the tree depth $T_{d}$, the branch length $B_{l}$, the branch angle $B_{a}$, the degree of randomness $D_{r}$, and the asymmetry of branches $A_{b}$ (see SI: Random trees generation). We defined a control group as a set of trees generated with predefined parameters ($T_{d}=5$, $B_{l}=10$, $B_{a}=\pi /4$, $D_{r}=10\%$, $A_{b}=0.0$) and independent random seeds. Each parameter was varied individually to generate groups of trees that differed from the control group in only one property. A tree is assigned to the group which is closer based on the comparison of the distances $d_{Bar}$ between the tree’s barcode and the barcodes of the trees in every group. This distance is used to construct a classifier based on a simple hierarchical clustering algorithm (Ward 1963). The accuracy of this classifier is defined as the percentage of successful trials.

**Fig. 3:**
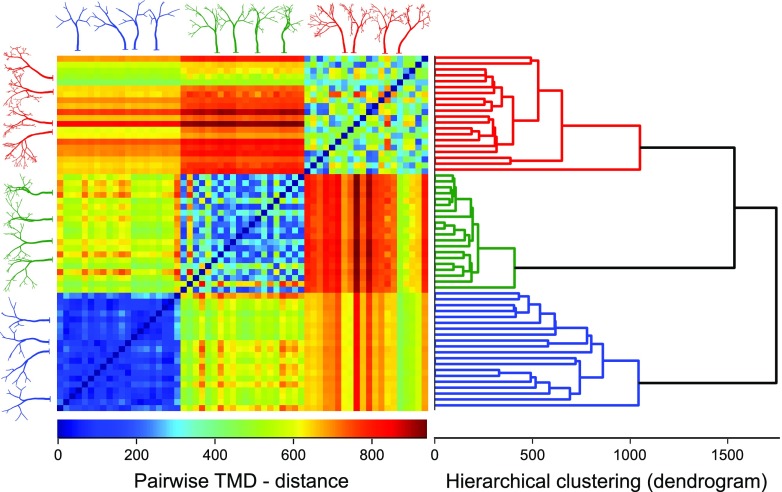
Topological analysis of artificial trees generated using a stochastic process. Three sets of trees are shown (only four individuals out of twenty for clarity). Each group differs from the others only in the tree depth. Each individual of the group is generated using the same tree parameters but a different random number seed. The TMD-distance of the trees allow their accurate separation into groups. The distance matrix indicates the existence of three groups which are identified with high accuracy by a simple dendrogram algorithm

We prove that this classifier efficiently separates groups of random trees that differ in their *tree depth* (Fig. 3), with an accuracy of $96\% \pm 3\%$ (see SI: Random trees grouping). In Fig. 3 the distance matrix indicates the existence of three distinct groups, and the corresponding clustering. The TMD of random trees generated by varying each of the other parameters $B_{a}$, $B_{l}$, $D_{r}$, $A_{b}$ are grouped with an accuracy of $88 \%$, $96 \%$, $99 \%$ and $100 \%$ respectively (see SI: Random trees grouping, Figs. S7-S11).

**Fig. 4:**
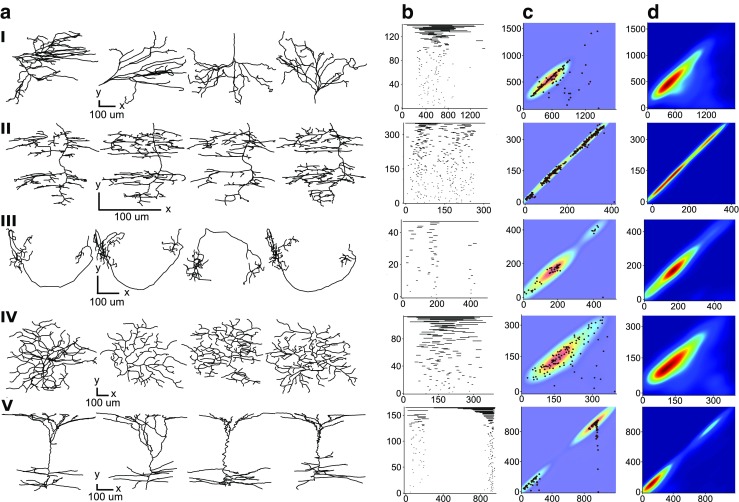
Topological comparison of neurons from different animal species. Each row corresponds to a species: (I) cat, (II) dragonfly, (III) fruit fly, (IV) mouse and (V) rat. Trees from several exemplar cells for each species are shown in the first column (**A**). Representative persistence barcodes for the cells in A are shown in the second column (**B**). The structural differences of the trees are clearly evident in these barcodes. II, III and V have clusters of short components, clearly distinct from the largest component, while I and IV have bars of a quasi-continuous distribution of decreasing lengths. Also, barcodes III, and V show empty regions between dense regions of bars, indicating the existence of two clusters in the morphologies, while barcodes I and IV are dense overall. The unweighted persistence image for each representative barcode in B and its superimposed persistence diagram are shown in the third column (**C**). By combining the persistence diagrams in (**C**) for several trees we can define an average unweighted persistence image (**D**) in order to study and quantify the structural differences between distinct morphological groups. The trees in the first row (cat) are more tightly grouped than those in the second row (dragonfly), and two clusters are visible in the dragonfly trees. Considering rows 1 and 4, the extension of the elliptical peak perpendicular to the diagonal line reflects the variance in the length-scale mentioned earlier for a single cell’s barcode. Note that the trees, barcodes, and unweighted persistence images are not shown to the same scale for clarity: see the scale bar in each case

Next, the TMD is used to quantify differences between neuronal morphologies. Neurons that serve distinct functional purposes exhibit unique branching patterns (Cuntz et al. 2007; Van Elburg and Van Ooyen 2010). In this study, we used cat, dragonfly, fruit fly, mouse and rat neuronal trees. The qualitative differences between the neuronal tree types are evident from the individual geometrical tree shapes (Fig. 4A) as well as the extracted barcodes (Fig. 4B). The regions of different branching density are visible in the average unweighted persistence images of each group (Fig. 4D). Since branching density is thought to be correlated with connection probability (Snider et al. 2010), we can identify the anatomical parts of the trees that are important for the connectivity of different cell types.

The performance of a supervised classifier trained on the unweighted persistence images (see SI: Supervised Classification, Classification of neuronal trees) of the TMD results is demonstrated by the grouping of neuronal trees from the different species, shown in Fig. 4. The neuronal trees of the five different species are accurately ($84\%$) separated into the original groups. We note here that the performance of this process is reliable ($>70\%$) even for small training sets that contain only $25\%$ of the whole dataset (see SI: Classification of neuronal trees).

**Fig. 5:**
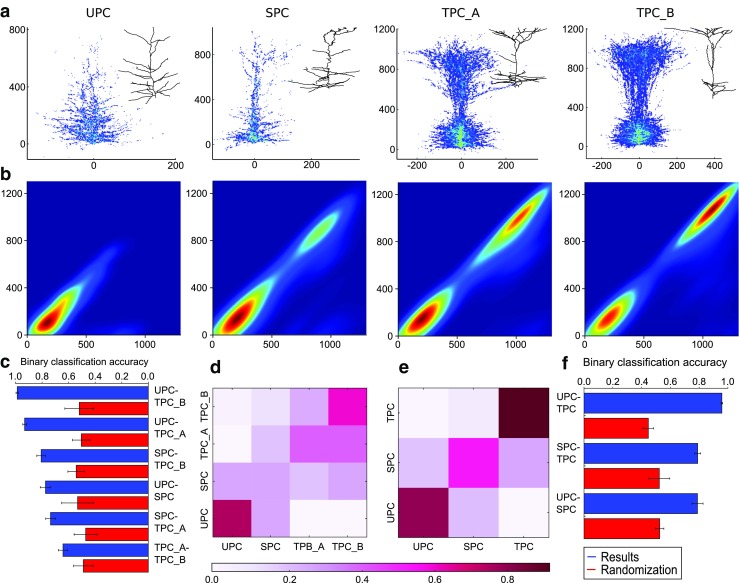
Comparison of the TMD of apical dendrite trees extracted from several types of rat pyramidal neuron. Four cell types are shown in (**A**): UPC, SPC, TPC-A, TPC-B (left to right). The morphological differences between these cell types are subtle, but the unweighted persistence images (**B**) clearly reveal them, particularly the presence of two clusters in the TPC-A and TPC-B cell types. From these unweighted persistence images we train a decision tree classifier on the expert-assigned groups of cells. The binary classification (**C**) and the confusion matrix (**D**) based on the TMD algorithm shows an overlap of TPC-A and TPC-B trees. When those two classes are merged (**E**, **F**) the separation between the remaining types is evident. This result shows that the unweighted persistence images objectively support the expert’s classification when the morphological differences between the classes are significant

We applied the TMD algorithm to a more challenging use case, because it is difficult for a non-expert to distinguish the different morphologies. While pyramidal cell (PC) morphologies (Fig. 5A) of the rat appear superficially similar, the unweighted persistence images (Fig. 5B) reveal fundamental morphological differences between them, related to the existence and the shape of the apical tuft. The apical tuft of PCs is known to play a key role in the integration of neuronal inputs through their synapses in higher cortical layers, and is therefore a key indicator for the functional role of the cell.

The separation of the PC trees into four groups cannot be justified based on purely morphological grounds, since there is no coherent difference between the branching patterns of TPC-A and TPC-B (Fig. 5C, D). On the contrary, the separation in three groups (UPC, SPC and TPC -the superset of TPC-A and TPC-B- Fig. 5E, F) is supported by TMD-based classifiers, by detecting the fundamental differences between their branching structures. Therefore, the TMD provides a solid benchmark test to objectively support or disprove proposed classification schemes.

Finally, the TMD algorithm can be used to assess the quality of any manually or automatically reconstructed neuron if a reference morphology is available. The best use case for this application is the datasets of BigNeuron (Peng et al. 2015), a community effort to advance single-neuron automatic reconstruction. The same stack of images of a scanned morphology is used for manual reconstruction (reference morphology) and for automatic reconstructions with a set of algorithms (test set). Due to the large number of reconstructions generated by the BigNeuron project, the analysis of the data requires a high-computational-performance algorithm. The linear complexity of the TMD makes it highly suitable for the analysis of this large dataset.

**Fig. 6:**
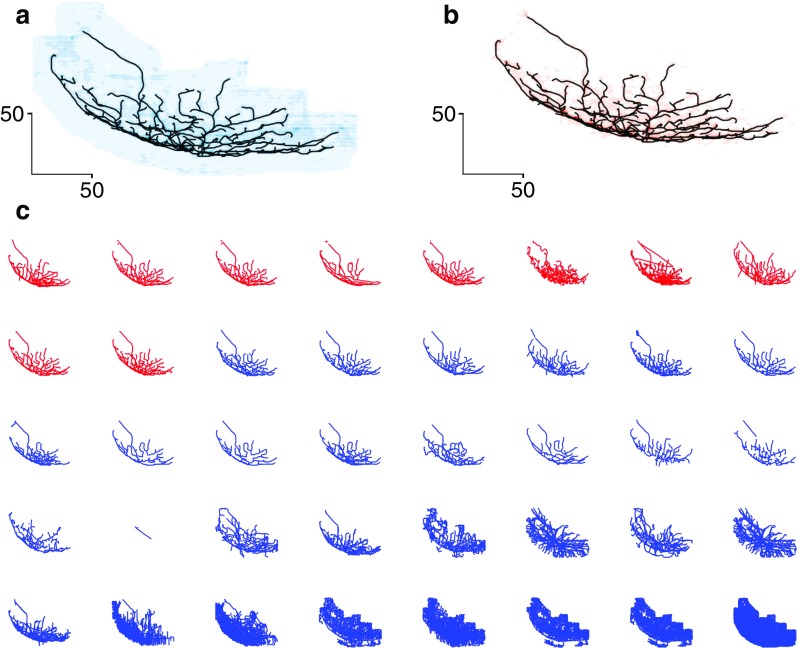
Comparison of the TMD of BigNeuron neuronal morphologies. An image stack is used for the manual reconstruction (reference neuron) and for the automatic reconstructions produced by a variety of community supplied algorithms. The results of each algorithm are illustrated in panel C, from best (top left) to worst (bottom right). The reference neuron (in black) is visualized against the density plot of all the automatically reconstructed neurons (A, in blue), and the density plot of the ten best automatic reconstructions (B, in red), ranked according to their TMD-distance from the reference neuron. A comparison between panels A and B shows that the density plot of the ten highest ranked automatic reconstructions closely matches the structure of the reference morphology

The automatic reconstructions were ranked based on their TMD-distance from the reference morphology. The TMD was able to accurately assess the quality of the automatic reconstructions, as presented in Fig. 6, as the similarity of the branching structure of the automatic reconstructions to the reference neuron decreases with the TMD-ranking. The density plot of all the automatic reconstructions Fig. 6A does not reproduce the shape of the reference morphology, as reconstruction errors are over-represented. On the contrary, the density plot of the ten TMD-best reconstructions closely matches the structure of the reference morphology.

## Discussion

The morphological diversity of neurons supports the complex information-processing capabilities of the nervous system. A major challenge in neuroscience has therefore been to reliably describe the shape of neurons. We have introduced here the Topological Morphology Descriptor, derived from principles of persistent homology. The TMD of a tree retains enough topological information to allow the systematic comparison between branching morphologies. Therefore, it provides a topological benchmark for the rigorous comparison of different structures and it could advance our understanding of the anatomy and diversity of the neuronal morphologies.

This technique can be applied to any rooted tree equipped with a function defined on its nodes. Further biological examples include botanic trees (Lopez et al. 2010), corals (Kruszyṅski et al. 2007) and roots of plants (Wang et al. 2009). The method is not restricted to trees in $\mathbb {R}^{3}$, but can be generalized to any subset *T* of a metric space *M*, with a base-point *R*. A persistence barcode can then be extracted using a filtration by concentric spheres in *M* centered at *R*, enabling us to efficiently study the shape of complex multidimensional objects.

While the static neuronal structures presented in this paper are biologically interesting themselves, our method could also be generalized to track the morphological evolution of trees. The topological study of the growth of an embedded tree could be addressed through Multidimensional Persistence (Carlsson and Zomorodian 2009), a new area of TDA, for which computational tools are currently being explored (Lesnick and Wright 2015; Gäfvert 2016). In this case the spherical filtration identifying relevant topological features of the tree could be enriched with a second filtration representing temporal evolution. This application could be useful in agriculture to study growing roots (Wang et al. 2009) and trees, and in neuroscience, to study neurons in the developing brain.

## Electronic supplementary material

Below is the link to the electronic supplementary material.

(PDF 3.37 MB)
