# Supplementary material for: A Topological Representation of Branching Neuronal Morphologies
Source: Neuroinformatics. 2017 Oct 3;16(1):3–13. doi: 10.1007/s12021-017-9341-1 (PMC5797226; doi:10.1007/s12021-017-9341-1)
Supplement: Supplementary file 1 — (PDF 3.37 MB) [file 12021_2017_9341_MOESM1_ESM.pdf]

Supporting Information: A topological representation of branching neuronal morphologies

Lida Kanari · Paweł Dłotko · Martina Scolamiero · Ran Levi · Julian Shillcock · Kathryn Hess · Henry Markram

1. Morphological clustering

Traditionally, the different morphological shapes of neurons have been qualitatively described based on visual inspection and quantitatively described based on morphometric parameters. Feature extraction results in significant loss of information, as the dimensionality of the data is significantly reduced. As a result, a limited set of selected features is not sufficient to capture the full complexity of the neuronal shapes. On the other hand, a large number of features will result in overfitting, since the correlated features are accounted for multiple times. In fact, the feature-based classification of neuronal trees strongly depends on the set of morphometrics that are used as input. Alternative sets of morphometrics result in different classifications [1] for the same set of cells.

In this section we illustrate the problems of this method with a simple example. In Fig 1 we present the results of the feature classification for a set of neuronal trees that belong in three distinct groups (axons, basal and apical

L. Kanari
Blue Brain Project, EPFL
E-mail: lida.kanari@epfl.ch

P. Dłotko
Department of Mathematics, Swansea University

M. Scolamiero
Laboratory for Topology and Neuroscience at the Brain Mind Institute, EPFL

R. Levi
Institute of Mathematics, University of Aberdeen

J. C. Shillcock
Blue Brain Project, EPFL

K. Hess
Laboratory for Topology and Neuroscience at the Brain Mind Institute, EPFL

H. Markram
Blue Brain Project, EPFL

dendrites). The data used for this grouping are given in Tables 1, 2, 3. The visual separation of the trees into three groups is presented in Fig S 1A. Even though two of the most important anatomical features, i.e., the total length and the total number of branches of the tree, are used, the resulting clustering does not correspond to the biological grouping (colormap in Fig S 1).

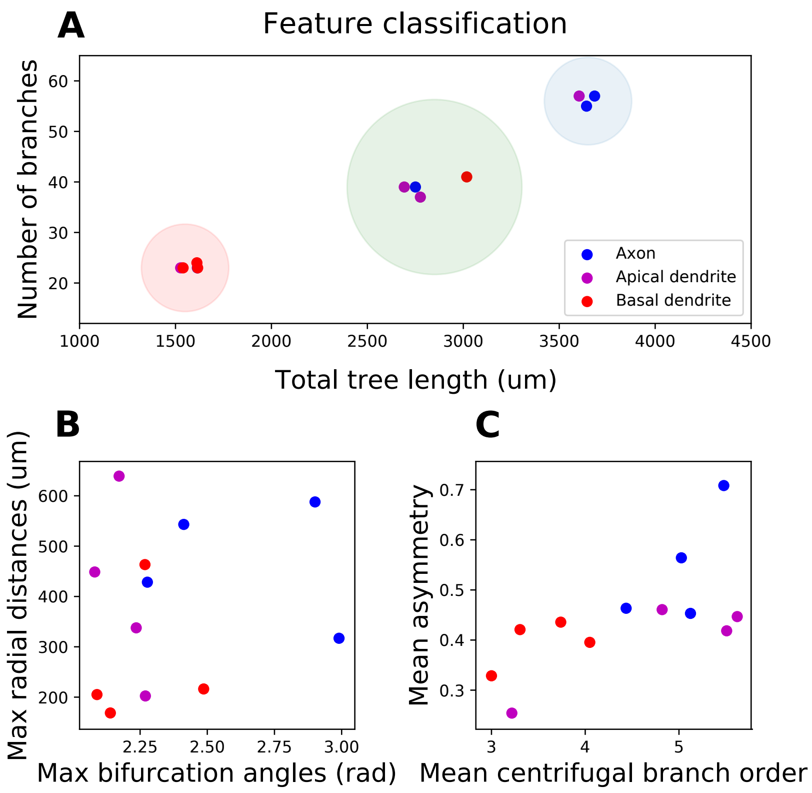

S 1: Visual separation of trees into groups according to selected morphological features. Three instances of the clustering are presented using different pairs of features (A: total tree length - number of branches, B: maximum branch angles - maximum radial distances, C: average branch order - average asymmetry); the results are not consistent. Inappropriate feature selection will therefore result in a grouping that does not correspond to the biological role of the three tree types (axons, apical and basal dendrites) as shown in all three cases.

Alternative feature pairs (maximum branch angles - maximum radial distances Fig S 1B, average centrifugal branch order [2]- average asymmetry [3] Fig S 1C) cannot reproduce the biological grouping or the initial classification (Fig S 1A). The results of the principal component analysis (PCA-decomposition) based on the six morphological features are presented in Fig S 2. We present the three first components with explained variance 96%. Even though six of the most significant morphological features were used for this

Table 1: Morphological features (Number of branches, Total length) extracted from the trees (axons, apicals, basals) presented in Fig S 1A.

Tree ID	Num branches	Total length(um)
Axon 1	57	3684.24
Axon 2	55	3642.24
Axon 3	39	2750.47
Axon 4	23	1614.04
Apical 1	57	3603.75
Apical 2	37	2776.20
Apical 3	39	2692.38
Apical 4	23	1526.87
Basal 1	41	3017.89
Basal 2	24	1611.49
Basal 3	23	1539.61
Basal 4	23	1615.44

Table 2: Morphological features (Max branch angles, Max radial distances) extracted from the trees (axons, apicals, basals) presented in Fig S 1B.

Tree ID	Max branch angles	Max radial distances
Axon 1	2.99	316.97
Axon 2	2.28	428.41
Axon 3	2.90	587.82
Axon 4	2.41	543.14
Apical 1	2.08	448.66
Apical 2	2.17	639.10
Apical 3	2.24	337.68
Apical 4	2.27	202.35
Basal 1	2.27	463.33
Basal 2	2.09	205.11
Basal 3	2.14	168.58
Basal 4	2.49	216.26

Table 3: Morphological features (Mean branch orders, Mean asymmetry) extracted from the trees (axons, apicals, basals) presented in Fig S 1C.

Tree ID	Mean centrifugal branch orders	Mean asymmetry
Axon 1	5.12	0.45
Axon 2	4.44	0.46
Axon 3	5.03	0.56
Axon 4	5.48	0.71
Apical 1	5.51	0.42
Apical 2	5.62	0.45
Apical 3	4.82	0.46
Apical 4	3.22	0.25
Basal 1	4.05	0.40
Basal 2	3.00	0.33
Basal 3	3.74	0.44
Basal 4	3.30	0.42

analysis, the three biological types of trees cannot be retrieved. This observation indicates that the feature based classification of neuronal trees is very sensitive to the selected features. As a result, a feature based classification is not reliable, as the appropriate feature set cannot be generalized across different groups.

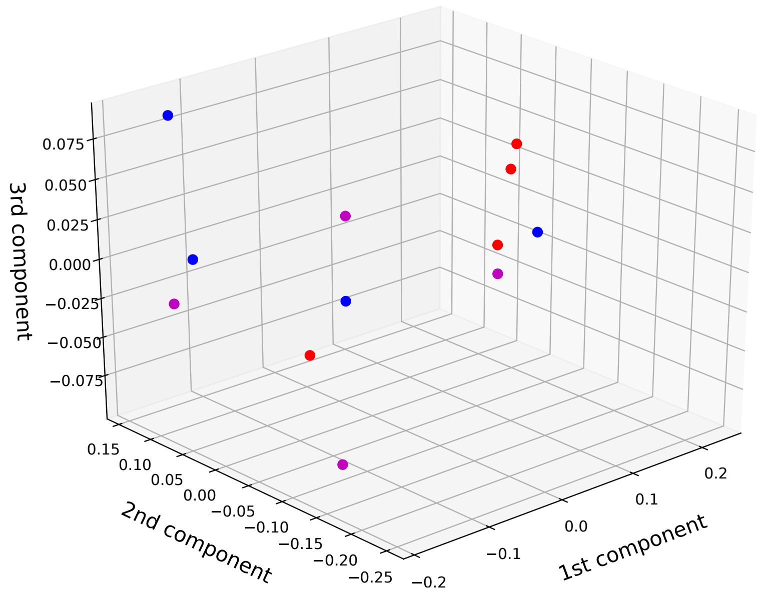

S 2: Principal component analysis on the normalized values of the six selected morphometrics of Fig S 1 (total tree length, number of branches, maximum branch angles, maximum radial distances, average branch order, average asymmetry). The PCA of the selected feature set is not able to distinguish the correct groups that correspond to the biological role of each tree, despite the different branching patterns that they present. Hence, inappropriate feature selection can indeed result in misclassification.

Demonstration of the TMD algorithm

The idea of the TMD algorithm is presented in Figs S 3A. The input of the TMD algorithm is a rooted tree with a function f defined on the set of nodes. In this example, the function f is the radial distance. The root, denoted by R, is shown in red, while the other nodes of the tree are labeled $a - i$. Note that the set of nodes consists of the branch points and the leaves. During the initialization of the algorithm, the leaves (a, c, e, g, h) are inserted into the list of active nodes, A. The algorithm then iterates over the members of A. The order of this process is not significant. Recall that the function v assigns to a node n of the tree the largest value of the function f on the leaves of the subtree with root at n (see Methods).

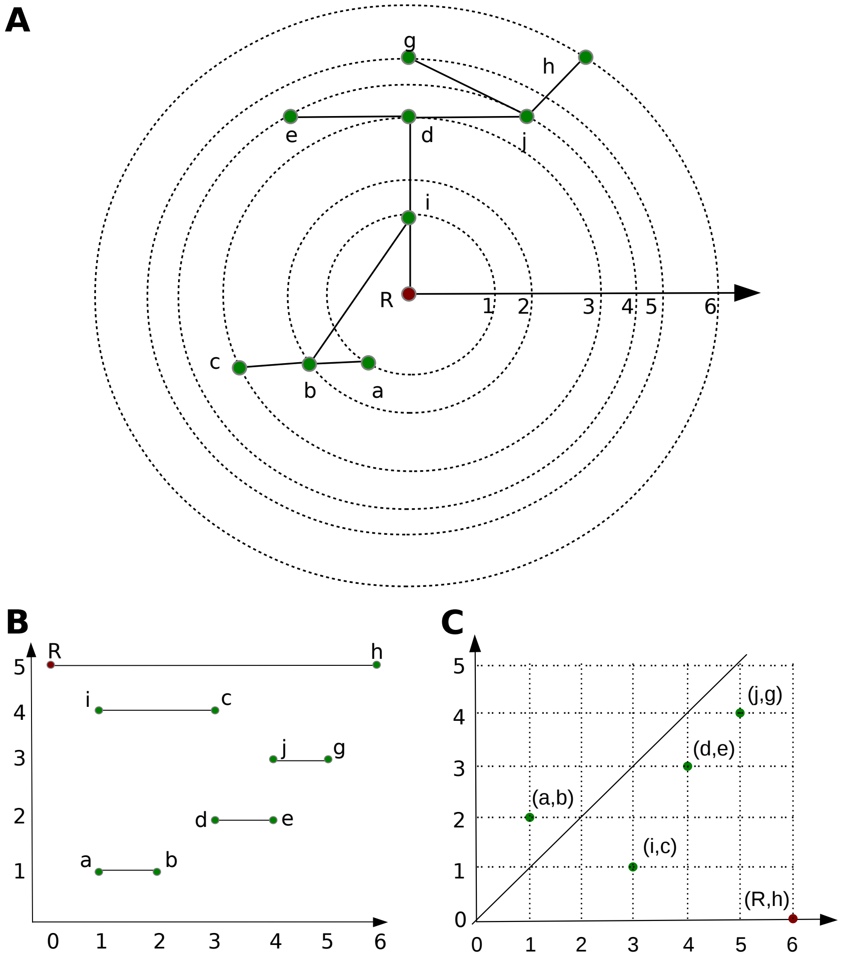

S 3: Demonstration of the TMD algorithm: A simple embedded rooted tree (A) is transformed with the TMD algorithm into the corresponding persistence barcode (B) and the equivalent persistence diagram (C). The root (R) is colored red, while the branch points and leaves are shown in green. The edges connecting corresponding pairs of points are presented by straight lines. The dashed circles are provided as a guide to the eye to indicate different levels of radial distances. The correspondence between the tree (A) and its extracted barcode (B) and its diagram (C) is given by the notation of the same nodes in both figures. Each bar in (B) represents the lifetime of a component. The positions of x-axis correspond to the circles in (A) while y-axis represents individual components, ordered according to their length. In (C) each point represents the birth and death time of a branch component in A.

The algorithm assigns the values of v on the leaves: $v(a) = f(a) = 1, v(c) = f(c) = 3, v(e) = f(e) = 4, v(g) = f(g) = 5, v(h) = f(h) = 6$. Consider the node a as the first element of the list A. The parent of a is b and its only other child is c. Since both a and c are in A, the algorithm orders the siblings

according to the values of function v. The older sibling is c and therefore $v(b) = v(c) = 3$. The interval $[1, 2]$ is added to the persistence barcode (Fig S 3B) TMD(T, f) representing the lifetime of the node a. This interval is equivalently represented as a point ab on the persistence diagram (Fig S 3C). Nodes a and c are removed from A, and b is added to A. The next vertex in the list A is e. The algorithm finds its parent, d, but this node is not processed further at this stage, since j, the sibling of d, is not in A. The next node to be processed is g. Both children g and h of j are in A. The oldest child is h and therefore $v(j) = v(h) = 6$. The interval $[5, 4]$, representing the lifetime of node g, is added to TMD(T, f). The node j is added to A, and both g and h are removed from A. The list of alive components then consists of b, e, j. The node b cannot be processed since its sibling d is not in A. The next node to be processed is therefore e, whose parent d has all of its children in A. In this case, the node with highest value of v is j, and therefore $v(d) = v(j) = 6$. The interval $[4, 3]$ is added to TMD(T, f). Then nodes e and j are removed from A, and d is added to A. The next node in A is b, whose parent is i. Since both children of i are now in A, the algorithm finds the older sibling, d and assigns $v(i) = v(d) = 6$. The interval $[3, 1]$ is added to TMD(T, f) and d, b are removed from A, while i is added to A. The only alive node is now i whose parent is the root R. The algorithm computes $v(R) = v(i) = 6$, i is removed from A and R is added in A. Since only the root R is alive, the while loop in the algorithm terminates. The last step adds the interval $[6, 0]$ to TMD(T, f), which represents the largest component of the tree.

2. Using alternative functions for the TMD algorithm

In the previous section, we applied the TMD algorithm with the radial distance as the filtration function f. Any alternative function f can be used, such as the path distance from the root, which should serve to reveal shape characteristics that are independent of the radial distance and thus not captured by this approach. The constraint of rotational invariance could also be relaxed by projecting the radial distance to a selected axis, to map the spherical filtration into an ellipsoidal one, in order to study the relation of a tree's spatial density to its embedding space.

Depending on the classification problem, alternative morphometrics could be more appropriate for the separation of trees in classes. For instance, the path distance Fig S 4A would be more appropriate to capture the differences between tortuous and straight trees, while the projected radial distance Fig S 4C can discriminate trees with different spatial distribution of branches. In Fig S 4 we present four variations of the TMD using different morphometrics (radial distance from the soma (A), path distance from the soma (B), projected to the axis towards the pia radial distance (C), branch orders). Each morphometric captures different properties of the branching structure. Those and other morphometrics could be combined in a multidimensional persistence diagram for the better discrimination of trees.

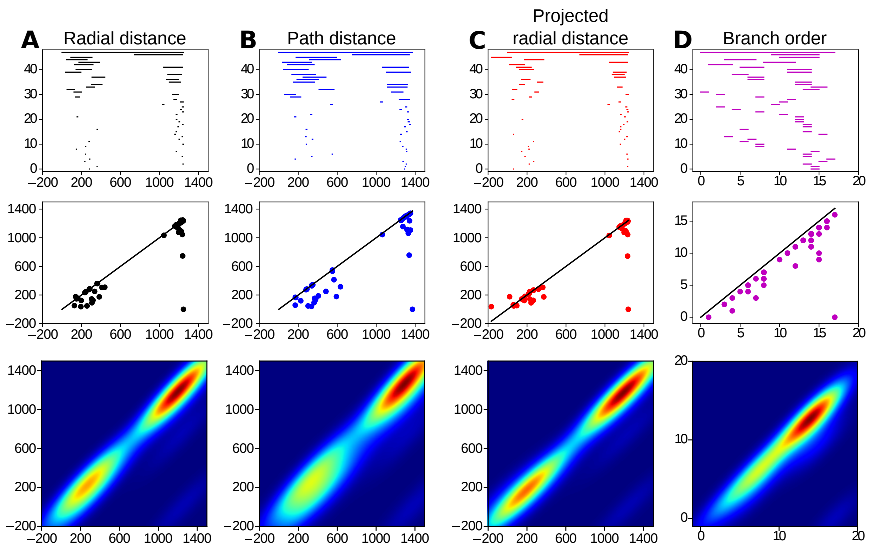

S 4: Demonstration of TMD algorithm for different morphological features. A. Radial distance from the soma. B. Path distance from the soma. C. Projected radial distance from the soma to the axis normal to the pia; this measurement can discriminate trees with different spatial distributions. D. Branch order; this measurement does not take into account the embedding in space, only the combinatorial branching patterns of the tree. Note the similarity among the three first morphometrics.

3. Definition of distances

In order to establish the comparison with the current literature we need to define a notion of distance between trees equipped with a real-valued function on their nodes, as well as a notion of distance between persistence diagrams.

3.1 Distances between persistence diagrams

Below we recall various representations of persistence diagrams and some notions of distance between them. We also provide a reference to software that computes the distances considered, when available. All of the metrics summarized below can be applied directly to the output of the TMD algorithm.

The most classical distances used in topological data analysis are the *bottleneck* and *Wasserstein* distances. Given a persistence diagram D, the points in the diagonal are "virtual" points, which have birth time equal to their death time. Therefore, we assume without loss of generality that a persistence diagram contain points in the diagonal with infinite multiplicity. Given two persistence diagrams D_1 and D_2, we construct a matching (i.e., a bijection) $\phi : D_1 \to D_2$ and define two numbers

$$B_\phi = \sup_{x \in D_1} d(x, \phi(x))$$

and

$$W^p_\phi = (\sum_{x \in D_1} d(x, \phi(x))^p)^{\frac{1}{p}},$$

where d is the standard Euclidean distance in $\mathbb{R}^2$. Note that B_ϕ is simply the longest distance that ϕ shifts a point in D_1, while $(W^p_\phi)^p$ is a sum of p-th powers of lengths of the line segments joining x and $\phi(x)$, for all x. The infimum of B_ϕ over all possible matchings is the *bottleneck distance* between D_1 and D_2. The infimum of W^p_ϕ over all possible matchings is the *$p-$ Wasserstein distance* between D_1 and D_2. Given two persistence diagrams D_1 and D_2, their bottleneck distance will be denoted by $d_B(D_1, D_2)$ and their $p-$Wasserstein distance by $W_p(D_1, D_2)$. One implementation of these distances is given in [13] and a faster approximation in [14].

A persistence diagram can also be represented by a persistence landscape, i.e., a piecewise linear function $\mathcal{L} : \mathbb{R} \times \mathbb{N} \to \mathbb{R}$. Given two persistence landscapes, we can compute the distance between them in $\mathcal{L}^p$ space [15]. The implementation is described in [16].

One can also encode persistence diagrams by unweighted persistence images as described in the main text. The idea is to apply a smoothing function, i.e., a Gaussian kernel, at every point of the diagram and then to discretize the distribution obtained into a pixel-based image. It is then straightforward to compute a distance between two unweighted persistence images, using common image-recognition techniques. A simplified version of this representation is used in the classification of morphological types of neurons in the experimental section of this paper. We are not aware of a publicly available implementation of this approach. An implementation is provided with the software of this paper.

3.2 Distances between trees

A classic metric to compare trees, the *edit distance* [8], is based on the transformation of one tree T_1 into another T_2 by a sequence of operations (deletion and insertion of vertices), each of which has a non-negative cost. The edit distance [8] between T_1 and T_2 is defined to be the infimum of the total cost of all possible transformations from T_1 to T_2. However, the edit distance is not relevant to our problem, since it does not involve geometric information about the tree structure and is known to be NP-complete [17].

An important notion of distance is the one between merge trees as defined in [18] and [19]. This distance is applied to merge trees of sublevel sets of functions. For a function $f : X \to \mathbb{R}$, where X is a metric space, the sublevel set at level $a \in \mathbb{R}$ is $\{x \in X | f(x) \leq a\}$. The differences captured by merge

trees are considerably more subtle than the differences captured by the persistent homology of the function's sublevel sets. The authors of [18] and [19] provide examples of pairs of simple merge trees T and T' that have the same persistence diagrams, but that are a nonzero distance apart. It is clear that in this particular case, the TMD would provide the persistent homology of the sublevel sets of the function. Therefore, by rescaling T and T', the difference between the distances used in those papers and the distances used in the current paper can get arbitrarily large.

Another relevant metric is the *persistence distortion distance* [12] between two graphs G_1 and G_2. To compute this distance one must calculate the shortest path distance from a fixed point to any other point in the tree for all $v_1 \in G_1$, denoted $P(G_1, v_1)$, and all $v_2 \in G_2$, denoted $P(G_2, v_2)$. Given the shortest paths, the persistence distortion is defined as the minimal bottleneck distance between the persistence diagrams in dimension zero of the superlevel sets of the distance functions $P(G_1, v_1)$ and $P(G_2, v_2)$. The persistence diagrams obtained in the process are conceptually very close to the diagrams we get from the TMD algorithm. In our case, we obtain a significant computational advantage from working with rooted trees, since there is always a unique path between every pair of vertices. Moreover a reasonable choice of initial vertices v_1 and v_2 from which to compute shortest paths is to take the root of the trees considered, given that this is the computational center of the neuron. In this case, the persistence diagram arising when computing the persistence distortion distance is the one we would get from the TMD algorithm when the function f is the path distance from the root. The computational cost of the distortion distance is considerable in the general case, but linear in our case. However, since the persistence distortion distance is based on the bottleneck distance, it suffers from that metric's limitations, i.e., the shortest components, which are important for the neuronal morphologies, are not taken into account. The code to compute persistence distortion distance is available here [20].

3.3 Distances between neurons

Strahler ordering [5], [6], a metric introduced for the study of a river's branching patterns, assigns a number to each branch of the tree, starting from the terminal branches (order 1) and increasing the ordering when branches of the same order merge. Strahler ordering analysis is similar to the TMD-algorithm because it starts from the terminal branches of the tree and proceeds from the outer branches towards the root. However, since the embedding of the tree is not considered, branches of different lengths are treated equally and their spatial distribution cannot be studied. The advantage of Strahler ordering is that the overall branching topology of the tree is captured in a single value and hence the comparison between trees is straightforward. However, depending on the branching structure, very complicated neuronal trees can be assigned low Strahler orders (for example a Hippocampus pyramidal cell can be of Strahler

order 4, see [6], Figure 3) so they are inseparable from simpler structures. This is once again due to the significant information loss of this analysis.

Sholl analysis [7] is a typical measurement used to study neuronal morphologies. It counts the number of segments that intersect with a set of equidistant spheres $\{S_0, S_1, S_2, ..., S_s\}$ of increasing diameters $\{0, sd, 2sd, ...\}$. Because of the high frequency of local fluctuations, the choice of the diameter step sd has a significant impact on the result of this analysis. While the Sholl analysis counts the number of components at each level, the persistence diagram of a tree T tracks the evolution of those components in space. As a result, the persistence diagram of a tree contains strictly more information than the Sholl analysis. In fact, the Sholl analysis can be retrieved from the TMD of a tree using a discretized version of distance d_{Bar}, which is defined in Methods. Similarly to the Sholl analysis the d_{Bar} distance encodes the number of components of the tree for a set of spheres of increasing diameters with a few significant differences. First, d_{Bar} does not depend on a choice of diameter step, so it is not subject to local fluctuations. In addition, the distance d_{Bar} counts the number of intersections of the branches of a tree with a sphere, as opposed to the segments that are counted in Sholl analysis. As a result, this distance is equivalent to a continuous version of Sholl analysis that processes the branches of the tree. This distance collapses the barcode structure into one dimension which results in significant information loss. As a result, it is not appropriate to distinguish subtypes of trees that express similar branching structures, such as subtypes of pyramidal cells (Figure 5).

A novel metric that is useful for distinguishing neuronal trees was proposed in [4]. *Blastneuron* focuses on the comparison of neurons based on the alignment of the branches by topology and path shapes after first defining similar neurons on the basis of their morphometrics. A set of morphological features is extracted from the trees, and the initial estimation of the distance between them is defined by the distance between the extracted features. An alignment algorithm is then applied to pairs of trees in order to identify local similarities. The local alignment requires the comparison of all pairs of branches, making the computation very expensive. This method is designed for the efficient matching of trees with highly similar structures, but the high variability within the groups of rat cortical neurons does not allow similar trees to be grouped together by local alignment, since local structures are often altered, depending on the location of the cells in the tissue.

The most recent advance in the field was made by *sequence representation* [9], an original encoding of trees as sequences of characters 'ACT' representing the local topology. Bifurcations are encoded on the basis of whether their children branch or terminate. Arborizing bifurcations (in which both child branches bifurcate) are encoded with the letter 'A', bifurcations with one bifurcating child and one terminating are encoded as 'C' and terminating bifurcations (with two terminating children) as 'T'. This method enables us to align different trees via Sequence-based Tree Alignment, which can be used for the assignment of a similarity score between trees, using cluster analysis. Furthermore, this method is useful for the generation of a consensus repre-

sentation [10] from a group of neurons that reveals the conserved structural properties of the corresponding trees. This technique is the closest existing method to the proposed TMD, since it reveals the topological properties that are persistent throughout a group of trees. However, the TMD takes into account the embedding of the tree in space preserving the relation between the short and long components of the tree. Furthermore, the TMD algorithm has a computational advantage over the highly computationally demanding sequence alignment techniques.

4. Stability of TMD

Let T denote a finite rooted tree with vertex set N containing a distinguished root R, which endows each edge of T with a natural orientation away from the R. Let $f : N \to \mathbb{R}$ be any function satisfying $f(n) > f(R)$ for all $n \neq R$ in N, i.e., f takes its lowest value at the root R. A pair (T, f), where T is a rooted tree (not assumed to be embedded in any ambient space) and f is a function satisfying the condition above, is referred to as a *TMD-pair*.

In this section we prove that the TMD algorithm that associates with a TMD-pair (T, f) a persistence diagram TMD(T, f) is robust under the type of perturbations of the tree T and the function f that are most likely to arise in the *reconstruction process*, i.e., the transformation of a physical tree-like object, such as a neuron, into input data for the TMD algorithm. We consider two types of reconstruction errors:

E1. error in measuring the exact placement of a node, and
E2. omission or addition of a small branch.

Errors of type **E2** may have the effect of changing the tree considered, which implies that the function f defined on its nodes takes on new values or loses a few of its previous values. Errors of type **E1** may affect the values of the function f on the nodes of the tree, though the abstract graph underlying the tree remains the same.

We now define four types of perturbations of TMD-pairs that will be considered admissible for our purposes. If T is a tree, then by *"adding a branch"* to T, we mean attaching a new branch to any node of T or adding a node to the interior of an existing branch of T and attaching a new branch to that node.

Definition 1 Fix a TMD-pair (T, f) and a real number $\epsilon > 0$. An elementary ϵ-perturbation of (T, f) is a TMD-pair (T', f') obtained from (T, f) by one of the following operations.

T1. $T = T'$, $f(R) = f'(R)$, and for all $n \neq R$, $|f'(n) - f(n)| < \epsilon$.
T2. T' is obtained from T by adding a branch at a node n of T, $f'(n) = f(n)$, and $|f'(n') - f(n)| < \epsilon$, where n' is the added leaf (univalent node). The restriction of f' to the nodes of T is equal to f.
T3. T' is obtained from T by adding an internal node n' on an existing edge in T, with incident nodes u and v, and a branch at n' with leaf n'', such

that $|f'(n') - f'(n'')| < \epsilon$, while $f'(n')$ lies between $f(u)$ and $f(v)$, or $|f'(n') - f(u)| < \epsilon$, or $|f'(n') - f(v)| < \epsilon$. The restriction of f' to the nodes of T is equal to f.

T4. T' is obtained from T by removing a branch with incident nodes n', n'', where n'' is a leaf, such that $|f(n') - f(n'')| < \epsilon$. The function f' is the restriction of f to T'.

A TMD-pair (T', f') is said to be an ϵ-perturbation of (T, f) if (T', f') is obtained from (T, f) by

i) performing operations of type **T1** on a subset of the set of nodes of T, and then
ii) performing a finite number of operations of types **T2**, **T3**, and **T4** on the resulting tree, such that every branch that is present in T' but not in T is a leaf, and the following condition holds.
   – If nodes $\{v_i\}_{i=1}^t$ are added via operations of type **T3** to a branch in T with incident nodes u and v, then the deviation from linear order of the values $f'(v_i)$ according to the position of the v_i on the branch is smaller than ϵ for every pair of adjacent nodes.

Let $\mathcal{P}_\epsilon(T, f) = \{(T', f') \mid (T', f') \text{ is an } \epsilon\text{-perturbation of } (T, f)\}$

Example 1 Let T be a rooted tree embedded in $\mathbb{R}^3$, and let f be the real-valued function that assigns to a node n in T its Euclidean distance to the root R. An elementary ϵ-perturbation of type **T1** corresponds to moving nodes in space by at most ϵ. Elementary perturbations of types **T2**, **T3** and **T4** correspond to removing branches from or adding branches to T, such that the distance between their nodes is at most ϵ. At the end of this section we observe that in fact any TMD-pair can be thought of as arising in this way.

The following definition is standard in the literature.

Definition 2 Let T be a rooted tree with root R. The depth of a node n in T is the number of edges in the unique path connecting n to R. The depth of a tree T is the maximum depth of a node in T.

A tree of depth 1 is said to be a *corolla*. Let T be a corolla with root R and leaves $l_1, \dots l_m$. Let m_i denote the multiplicity of the value $f(l_i)$, for $1 \leq i \leq k$, where $l_1, ..l_k$ are the leaves on the function f assumes distinct values. The persistence diagram associated to (T, f) through the TMD algorithm has the form

$$TMD(T, f) = \Big\{ \big(f(l_i), f(R)\big)^{m_i} \ \mid \ 1 \leq i \leq k \Big\} \cup D,$$

where $(x, y)^j$ denotes the point (x, y) with multiplicity j, and D is the diagonal. If the values of function f are non-negative, then it suffices to consider D as the first quadrant diagonal.

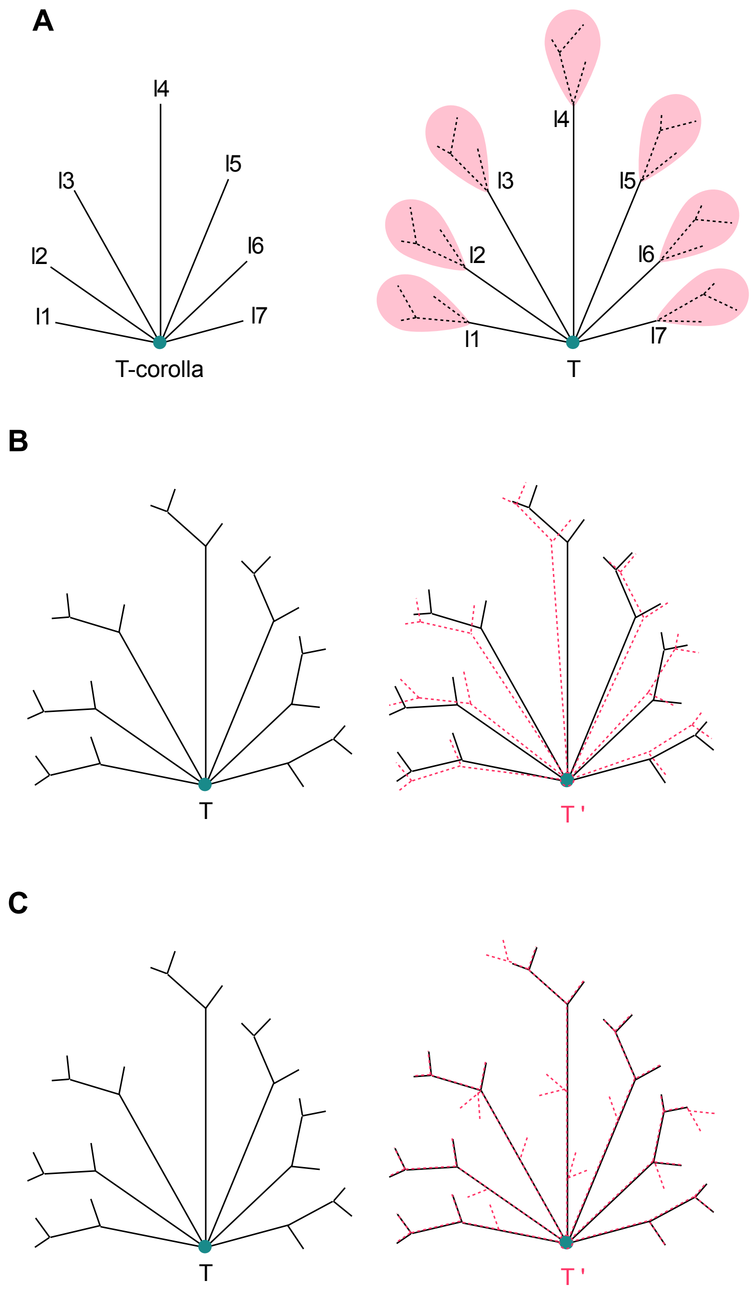

S 5: A. Illustration of a T-corolla tree and an associated tree with basis the corolla tree. B.-C. Small perturbations in the structure of the tree: B) type E1: the node's h position has been moved by ϵ to the node h'. This figure illustrates one of the possible perturbations, that will be used for the proof of stability of the TMD algorithm. C) type E2: ϵ small branches have been added to the tree. This figure illustrates one of the possible perturbations, that will be used for the proof of stability of the TMD algorithm.

Let T be any tree of depth h and root R. For a node $n \in T$, recall that T_n denotes the subtree of T starting at n, considered as a rooted tree with root n. In particular $T_R = T$ and for any $n \neq R$, T_n is a tree of depth strictly less than h.

Let $n_1, \dots n_m$ be the nodes in T of depth 1 (i.e., the children of R). For every i, let

$$b_i = \max\{x \mid (x, y) \in TMD(T_{n_i}, f)\}$$

(i.e., b_i is the largest value of f on a node of T_{n_i}). Then for each i the point $(b_i, f(n_i))$ is in the persistence diagram TMD(T_{n_i}, f), and one easily observes that

TMD(T,f)= $\big\{$ (b$_i, f(R)) \mid 1 \leq i \leq m\big\} \sqcup \coprod_{i=1}^m TMD(T_{n_i}, f) \setminus \big\{(b_i, f(n_i)) \mid 1 \leq i \leq m\big\}$

We can now establish the stability of the TMD algorithm with respect to bottleneck distance under ϵ-perturbations of TMD-pairs.

Theorem 1 *Let (T, f) be a TMD-pair, and let $\epsilon > 0$. If (T', f') is an ϵ-perturbation of (T, f), then*

$$d_B\big(TMD(T, f), TMD(T', f')\big) \leq 3\epsilon.$$

Proof The proof proceeds by induction on the depth of T, separating the cases in which T' is obtained from T through operations of type **T1**, **T2**, or **T3**. Since any set of operations of type **T4** reverses a corresponding set of operations of types **T2** and **T3**, and since bottleneck distance is a metric (and hence symmetric), the effect of perturbations of type **T4** will be discussed only briefly.

Perturbations of type **T1**.

If (T', f') is a TMD-pair obtained from (T, f) by perturbations of type **T1**, then the depth of T is equal to the depth of T'. For every node n in T, we denote by n' the corresponding node in T'. To compute an upper bound on the bottleneck distance between TMD(T, f) and TMD(T', f'), we construct a specific type of matching between their persistence diagrams. Recall that

TMD(T, f)= $\big\{$ $\big($b$_i, f(R)\big) \mid 1 \leq i \leq m\big\} \sqcup \coprod_{i=1}^m TMD(T_{n_i}, f) \setminus \big\{\big(b_i, f(n_i)\big) \mid 1 \leq i \leq m\big\}$

and

TMD(T', f')= $\big\{$ $\big($b'$_i, f'(R')\big) \mid 1 \leq i \leq m\big\} \sqcup \coprod_{i=1}^m TMD(T'_{n'_i}, f') \setminus \big\{\big(b'_i, f'(n'_i)\big) \mid 1 \leq i \leq m\big\}$

We show by induction on the depth of T that there exists a matching between TMD(T, f) and TMD(T', f') such that $\big(b_i, f(R)\big)$ is matched with $\big(b'_i, f'(R')\big)$ for every i and such that the L_∞-distance between each pair of matched points is less than ϵ, from which we deduce that the bottleneck distance bewteen TMD(T, f) and TMD(T', f') is also less than ϵ.

For the base step of the induction we consider a corolla T, with root R and leaves $l_1, \ldots, l_m$, whence T' is also a corolla with root R' and leaves $l'_1, \ldots, l'_m$. It follows that

$$\text{TMD(T,f)}= \Big\{\big(\text{u}_i, f(R)\big)^{m_i} \ \mid \ 1 \leq i \leq k\Big\} \cup D,$$

and

$$\text{TMD(T',f')}= \Big\{\big(\text{u'}_i, f'(R')\big)^{m'_i} \ \mid \ 1 \leq i \leq k'\Big\} \cup D,$$

where $\{u_i \mid 1 \leq i \leq k\}$ is the set of values of f on the nodes of T (other than the root R), and m_i is the multiplicity of u_i, for $1 \leq i \leq k$, while $\{u'_i \mid 1 \leq i \leq k'\}$ is the set of values of f' on the nodes of T' (other than the root R'), and m'_i denotes the multiplicity of the value u'_i, for $1 \leq i \leq k'$.

Condition **T1** implies that $|f(l_i) - f'(l'_i)| < \epsilon$ for all $1 \leq i \leq m$ and $|f(R) - f'(R')| < \epsilon$ and thus the L_∞-distance between the points $\big(f(l_i), f(R)\big)$ and $\big(f'(l'_i), f'(R')\big)$ is less than ϵ. Matching $\big(f(l_i), f(R)\big)$ with $\big(f(l'_i), f'(R')\big)$ for every $1 \leq i \leq m$, we see that ϵ is an upper bound on the bottleneck distance between TMD(T, f) and TMD(T', f') in this case, i.e.,

$$d_B\big(TMD(T, f), TMD(T', f')\big) < \epsilon.$$

The constructed matching is of the desired type.

Suppose now that the inductive hypothesis holds for all TMD-pairs (T, f), where T is a tree of depth less than h, and all $(T', f') \in P_\epsilon(T, f)$ obtained by perturbations of type **T1**. Let (T, f) be a TMD-pair where T is a tree of depth h. Assume that $(T', f') \in P_\epsilon(T, f)$ is obtained by perturbations of type **T1** from (T, f).

For each $1 \leq i \leq m$, let

$$\begin{aligned} C &= \big\{\big(b_i, f(R)\big) \mid 1 \leq i \leq m\big\}, \\ C' &= \big\{\big(b'_i, f'(R')\big) \mid 1 \leq i \leq m\big\}, \\ D_i &= TMD(T_{n_i}, f) \setminus \big\{\big(b_i, f(n_i)\big)\big\}, and \\ D'_i &= TMD(T'_{n'_i}, f') \setminus \big\{\big(b'_i, f'(n'_i)\big)\big\}. \end{aligned}$$

Matchings between D_i and D'_i for every i and a matching between $C \cup D$ and $C' \cup D$ together give rise to a matching between TMD(T, f) and TMD(T', f'), from which we can compute an upper bound on $d_B\big(TMD(T, f), TMD(T', f')\big)$.

Since $(T'_{n'_i}, f')$ is an ϵ-perturbation of (T_{n_i}, f) of type **T1** for all i, and each T_i is of depth less than h, the inductive hypothesis implies that for all i, there

is a matching between $\mathrm{TMD}(T_{n_i}, f)$ and $\mathrm{TMD}(T'_{n'_i}, f')$ such that $\big(b_i, f(n_i)\big)$ is matched with $\big(b'_i, f'(n'_i)\big)$ and such that the L_∞-distance between each pair of matched points is less than ϵ. By removing the matched pairs of points $\big(b_i, f(n_i)\big)$ and $\big(b'_i, f'(n'_i)\big)$, we obtain a matching between D_i and D'_i such that the L_∞-distance between every pair of matched points is less than ϵ. Moreover, the argument for the corolla case shows that there is a matching between $C \cup D$ and $C' \cup D$ that matches $\big(b_i, f(R)\big)$ with $\big(b'_i, f'(R')\big)$ for every i and such that the L_∞-distance between every pair of matched points is less than ϵ. The union of these two matchings gives rise to the desired matching between $\mathrm{TMD}(T, f)$ and $\mathrm{TMD}(T', f')$ that satisfies the inductive hypothesis. In particular,

$$\mathrm{d}_B\big(TMD(T,f), TMD(T',f')\big) \leq \max\Big(\big\{d_B\big(D_i, D'_i\big) \mid 1 \leq i \leq m\big\} \cup \{d_B(C, C')\}\Big) < \epsilon.$$

Perturbations of type **T2**.

Let $(T', f') \in P_\epsilon(T, f)$ be a TMD-pair obtained from (T, f) by perturbations of type **T2**. To set our notation, let $\{n_i\}_{i=1}^m$ denote the set of all nodes in T different from the root R. Let $\{n_i\}_{i=1}^r$ $(r \leq m)$ denote the nodes where new branches were added. For each $1 \leq i \leq r$, let $\{u_{i,k}\}_{k=1}^{q_i}$ denote the new nodes resulting from adding new branches at the node n_i. Finally let, $\{z_s\}_{s=1}^n$ denote the nodes added to T' as a result of adding branches at the root R. Thus the nodes in T' are

$$\{\mathrm{R}, \mathrm{n}_1, \ldots, n_r, n_{r+1}, \ldots, n_m\} \cup \{u_{i,k} \mid 1 \leq k \leq q_i, 1 \leq i \leq r\} \cup \{z_s \mid 1 \leq s \leq n\}.$$

With this notation, Condition **T2** ensures that $f(R) = f'(R)$, and

- for all $1 \leq i \leq m$, $f'(n_i) = f(n_i)$, and for all $1 \leq i \leq r$ and $1 \leq k \leq q_i$, $|f'(u_{i,k}) - f'(n_i)| < \epsilon$, and
- for all $1 \leq s \leq n$, $|f'(R) - f'(z_s)| < \epsilon$.

As in the previous case, the proof is carried out by induction: we prove the statement first in the case where T is a corolla, and then move on to the general case.

Assume T is a corolla. The persistence diagram for (T, f) has the form:

$$\mathrm{TMD(T, f)} = \coprod_{i=1}^m (f(n_i), f(R)) \cup D,$$

where D is the diagonal. On the other hand, the persistence diagram for (T', f') has the form:

$$\mathrm{TMD(T', f')} = \coprod_{i=r+1}^m \Big(f'(n_i), f'(R)\Big) \sqcup \coprod_{i=1}^r \Big(f'(u_{i,k_i}), f'(R)\Big) \sqcup L \sqcup \coprod_{s=1}^n \Big(f'(z_s), f'(R)\Big) \cup D,$$

where for each $1 \leq i \leq r$, u_{i,k_i} is a node on which f' obtains a maximal value among all nodes $\{u_{i,k}\}_{k=1}^{q_i}$, and L is a collection of points of the form $(n_i, u_{i,j})$ for those $j \neq k_i$ such that $f'(n_i) > f'(u_{i,j})$, and $(u_{i,j}, n_i)$ for $j \neq k_i$ such that $f'(n_i) < f'(u_{i,j})$. There is an obvious matching between the sets

$\coprod_{i=1}^{m}(f(n_i), f(R))$ in $\mathrm{TMD}(T, f)$ and

$\coprod_{i=r+1}^{m}\Big(f'(n_i), f'(R)\Big) \sqcup \coprod_{i=1}^{r}\Big(f'(u_{i,k_i}), f'(R)\Big)$ in $\mathrm{TMD}(T', f')$,

and the distance between any pair in this matching is bounded above by ϵ by Condition **T2**. All other points are at L_∞-distance at most ϵ from the diagonal. Hence matching those points to the diagonal gives an upper bound of ϵ on the bottleneck distance in this case.

For the induction step, let T be a tree of depth h with root R, where the nodes of depth 1 are denoted $l_1, \ldots, l_m$. For each $1 \leq i \leq m$, let T_{l_i} denote the subtree of T with root l_i. Let $x_i = T_{l_i} argmax f$ for each i. Let $TMD_0(T_{l_i}, f)$ denote the sub-diagram of $\mathrm{TMD}(T_{l_i}, f)$ consisting of all points except the unique one with $f(l_i)$ as its y-coordinate. The persistence diagram for T is of the form

TMD(T, f)= $\coprod_{i=1}^{m} TMD_0(T_{l_i}, f) \sqcup \coprod_{i=1}^{m}\big(f(x_i), f(R)\big) \sqcup D.$

Let T' be a rooted tree obtained from T with operations of type **T2**. For each i, let T'_{l_i} denote the subtree of T' with root l_i. As above,

TMD(T', f') = $\coprod_{i=1}^{m} TMD_0(T'_{l_i}, f') \sqcup \coprod_{i=1}^{m}\big(f'(y_i), f'(R)\big) \sqcup$
$\coprod_{s=1}^{n}\big(f'(z_s), f'(R)\big) \sqcup D,$

where $y_i = T'_{l_i} argmax(f')$ for each $1 \leq i \leq m$.

Notice that if D_1, D'_1, D_2, D'_2 are persistence diagrams such that $d_B(D_i, D'_i) \leq \delta$ for some $\delta > 0$ and for $i = 1, 2$, then $d_B(D_1 \sqcup D_2, D'_1 \sqcup D'_2) \leq \delta$. This observation and the induction hypothesis together show that

$\mathrm{d}_B(\coprod_{i=1}^{m} TMD_0(T_{l_i}, f), \coprod_{i=1}^{m} TMD_0(T'_{l_i}, f')) \leq \epsilon.$

Clearly, $y_i - x_i \leq \epsilon$ for each $1 \leq i \leq m$. Thus it follows that matching the points $(x_i, f(R))$ and $(y_i, f'(R))$ for each $1 \leq i \leq m$ does not increase the distance between the corresponding sub-diagrams. Finally notice that each point of the form $(f'(z_s), f'(R))$ is of L_∞-distance at most ϵ from the diagonal. Putting these observations together we conclude that

$\mathrm{d}_B(TMD(T, f), TMD(T', f')) \leq \epsilon,$

as claimed.

Perturbations of type **T3**.

Let $(T', f') \in P_\epsilon(T, f)$ be a TMD-pair obtained from (T, f) by perturbations of type **T3**. To set our notation for this case, let $\{v_j\}_{j=1}^t$ denote the new (internal) nodes added to T, i.e., the v_j are the nodes in T' where a branching point occurs that is not present in T. For each $1 \leq j \leq t$, let $\{w_{j,l}\}_{l=1}^{p_j}$ denote the new nodes resulting from adding branches at v_j.

Condition **T3** ensures that $f(R) = f'(R)$ and that the following statements hold.

- For all $1 \leq j \leq t$, $f'(v_j)$ is either an intermediate value between the values of f on the nodes incident to the edge along which v_j was added, or $f'(v_j)$ is no more than ϵ away from the value of f on at least one of those nodes.
- For all $1 \leq j \leq t$, and all $1 \leq l \leq p_j$, $|f'(w_{j,l}) - f'(v_j)| < \epsilon$.

Notice also that the values of f' on new nodes added on a single branch in T satisfy the extra linear ordering condition in Definition 1.

Once more, we start by assuming T is a corolla. As before, in this case,

$$TMD(T, f) = \coprod_{i=1}^{m} (f(n_i), f(R)) \cup D,$$

where D is the diagonal. For each $1 \leq i \leq m$, let e_i denote the i-th branch in T, and let e_i' denote the branch of T' corresponding to e_i. It follows that e_i' either is identical to e_i or contains one or more new branching points. Notice that $\mathrm{TMD}(T, f) = \coprod_{i=1}^{m} \mathrm{TMD}(e_i, f|_{e_i}) \cup D$, and similarly that $\mathrm{TMD}(T', f')$ $= \coprod_{i=1}^{m} \mathrm{TMD}(e_i', f|_{e_i'}) \cup D$. Hence it suffices to prove the claim for $m = 1$, i.e., when T is a corolla with exactly one leaf.

Let T consist of the root R and a node n with a single edge between them. Let $\{v_j\}_{j=1}^t$ denote the internal nodes added in T', and let $\{w_{j,l}\}_{l=1}^{p_j}$ denote the leaves added at v_j. For nodes v_j such that $f'(v_j)$ is not intermediate between $f(n)$ and $f(R)$, condition **T3** guarantees that the value of f' on those nodes and their branches is at most 2ϵ away from $f(n)$. Indeed, notice first that $f'(v_j)$ cannot be smaller than $f(R)$, by hypothesis. Hence the only way for $f'(v_j)$ not to be intermediate is to have $f'(v_j) > f(n)$. If this is the case, then $|f'(w_{j,l}) - f(n)| < 2\epsilon$. On the other hand, for nodes v_j such that $f'(v_j)$ is an intermediate value between $f(n)$ and $f(R)$, the contribution of an added leaf with end node $w_{j,l}$ to $\mathrm{TMD}(T', f')$ is easily seen to be L_∞-distance at most ϵ from the diagonal. Thus, let u be a node in T' such that $f'(u)$ is maximal (possibly $u = n$). Then the point $(f'(u), f'(R))$ can be matched with $(f(n), f(R))$. It is now easy to observe that all remaining points in $\mathrm{TMD}(T', f')$ are of L_∞-distance at most ϵ from the diagonal, and hence can be matched with diagonal points, so that the claim for the corolla follows.

The induction step now follows very similarly to the case of perturbations of type **T2**. Hence in this case we obtain once more

$$d_B(TMD(T, f), TMD(T', f')) < 2\epsilon.$$

Perturbations of type **T4**.

Let $(T', f') \in P_\epsilon(T, f)$ be a TMD-pair obtained from (T, f) by perturbations of type **T4**. Reversing the roles, (T, f) is a TMD-pair obtained from (T', f') by perturbations of type **T2** and **T3**. Hence by the discussion of perturbations of these types

$$d_B(TMD(T, f), TMD(T', f')) < 2\epsilon.$$

Conclusion.

We are now ready to complete the proof of the theorem. Notice that perturbations of types **T2**, **T3**, and **T4** can be performed on a TMD-pair (T, f) simultaneously without any complications. Hence if (T', f') is a TMD-pair that results from (T, f) by applying these operations, then the bottleneck distance between the corresponding persistence diagrams is bounded above by 2ϵ. Adding perturbations of type **T1** and using the triangle inequality for bottleneck distance, we conclude that

$$d_B(TMD(T, f), TMD(T', f')) \leq 3\epsilon$$

for all $(T', f') \in \mathcal{P}_\epsilon(T, f)$.

Note that errors in the connectivity of the tree are not considered. When the connectivity is modified, the new tree T^* will have a different topology. Therefore it is not possible to ensure that T and T^* will be ϵ-close and as a consequence $TMD(T)$ and $TMD(T^*)$ are not restricted to be ϵ-close.

A geometric interpretation.

We finish the section by observing that Example 1 is generic, in the sense that every TMD-pair (T, f) can be thought of as a rooted tree embedded in $\mathbb{R}^3$, with f the function given by radial distance from the root. Indeed, since we assume that $f(R)$ is the absolute minimal value of f on the nodes of T, there is no loss of generality in assuming that $f(R) = 0$. Since the set of nodes of the T is finite, the function f takes on finitely many values $0 < a_1 < \cdots < a_r$. Identify R with the origin in $\mathbb{R}^3$, and embed the set $f^{-1}(a_i)$ into the sphere of radius a_i about the origin for each i. Connect by a straight line each pair of points corresponding to nodes in T that are connected by an edge. Compactness of a finite union of line segments allows the transformation of this into an embedding by small perturbations, without moving points off of the sphere of radius a_i. The function f is now given by radial distance from the origin.

5. Random trees

5.1 Random tree generation

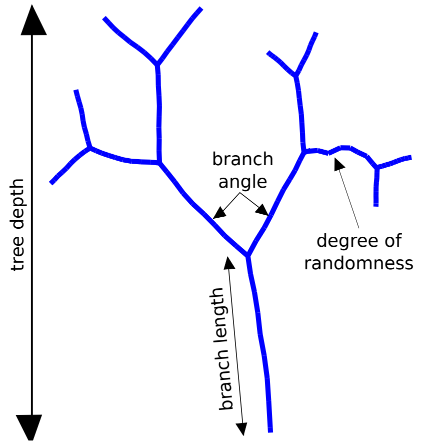

S 6: Random tree generation. Definition of growth parameters of artificial random trees: each tree is a perfect binary tree, which consists of branch points and leaves. A random walk defines the edges that connect pairs of points on the tree. The order of a branch is defined as the number of bifurcations between the branch point (or leaf) and the root. The tree depth is the maximum branch order of a tree. The branch length is the length of each edge. The branch angle defines the bifurcation angle between two children of a branch point. The degree of randomness indicates if the edge is a straight line or a simple random walk.

The random trees that were used for testing the TMD algorithm's performance were generated with software developed within the Blue Brain Project (BBP). Each tree consists of branches, i.e., paths between two branch points, which are generated based on a simple random walk (SRW, [21]) in $\mathbb{R}^3$. The position of the walk at each step is given as a weighted sum of a predefined direction d_n and a simple random walk Ψ:

$$X_{n+1} = X_n + w_s \cdot ((1 - D_r) \cdot d_n + D_r \cdot \Psi),$$

where w_s is the step size, and D_r defines the randomness of each step and Ψ is a random vector in $\mathbb{R}^3$ sampled from a uniform distribution. For $D_r = 0$ the branch is a straight line, while for $D_r = 1$ the branch is a SRW. The number of steps is given by the preselected branch length B_l. Once the number of steps is reached, the tree bifurcates, i.e., two new branches are created. The angle between the initial points of the branches is defined by the branch angle B_a. The tree generated this way is a perfect binary tree, i.e., every leaf has the same depth, since new branches are added at every branch point until the preselected tree depth T_d (i.e., the maximum number of edges in the unique path from a leaf to the root R) is reached. The total number of branches in the

tree is then $2^{T_d} - 1$. For example, the tree in Fig S 6 has $T_d = 4$ and consists of $2^4 - 1 = 15$ branches.

This set of parameters $\{T_d, B_l, B_a, D_r\}$ defines the global properties of the tree. Random trees that are generated with the same set of parameters share common morphometric properties, but have unique spatial structures, due to the stochastic component of the growth. This allowed us to check the effectiveness of the algorithm at identifying sets of trees that have been generated with the same input parameters $\{T_d, B_l, B_a, D_r\}$ and that differ only in the random seed. Random trees constructed with the described algorithm can intersect geometrically, even though the probability of this event is very low. However, for the random tree generation, the connectivity is obtained from the branches of the tree and therefore even if branches intersect geometrically, no cycle will be created in the tree.

5.2 Grouping random trees

We defined a control group as a set of trees generated with fixed parameters ($T_d = 5$, $B_l = 10$, $B_a = \pi/4$, $D_r = 10\%$) but independent random seeds. Then, we varied each parameter individually to generate groups of trees that differed from the control group in only one property. For all trees we extracted the persistence barcode using the TMD algorithm. The assignment of a tree to a group based on the comparison of the distances d_{Bar} between the tree's barcode and the barcodes of the trees in every group constitutes one trial. The trial is successful if the tree is correctly assigned to its original group. The performance of the TMD-based classifier in separating groups of trees generated with different values for each of the described parameters is summarized in Table 4. We cross-validate our method by generating 100 trees for each group, divided into 5 subsets of 20 trees. The standard deviation in Table 4 shows the statistical significance of our results.

Table 4: Summary of accuracy results for the classification of random trees.

$T_d : (4, 6, 8)$	$B_a : (\frac{\pi}{4}, \frac{\pi}{2}, \pi)$	$B_l : (5, 10, 30)$	$D_r : (0.1, 0.5, 0.8)$	$A_b : (0.0, 0.3, 0.9)$
$96 \pm 3\%$	$88 \pm 9\%$	$96 \pm 4\%$	$99 \pm 1\%$	$100 \pm 0\%$

The variation of the previous parameters includes only quantitative morphological features. All the generated trees are binary trees. In order to assess the performance of the TMD algorithm at grouping trees with different asymmetries, we generated trees with the same morphological features: number of terminal branches (16), branch lengths(100um), branch angles($\pi/3$) and degree of randomness(0.1) but different degree of asymmetry as defined in [3]. Trees with different degree of asymmetry express different topology of their branching patterns, the probabilities of which are described in [3]. The results of this analysis are presented in Fig S 11 for asymmetries of $0.0, 0.3, 0.9$.

The influence of each morphological feature (tree depth, branch length, branch angles, degree of randomness and degree of asymmetry) on the corresponding persistence barcode is described in detailed in Figures S 7 - S 11. The TMD-based classifier is able to distinguish the variation of all five parameters with significantly high accuracy. This indicates that the TMD-based distance is effectively separating artificial random trees that differ in one of the described morphological properties.

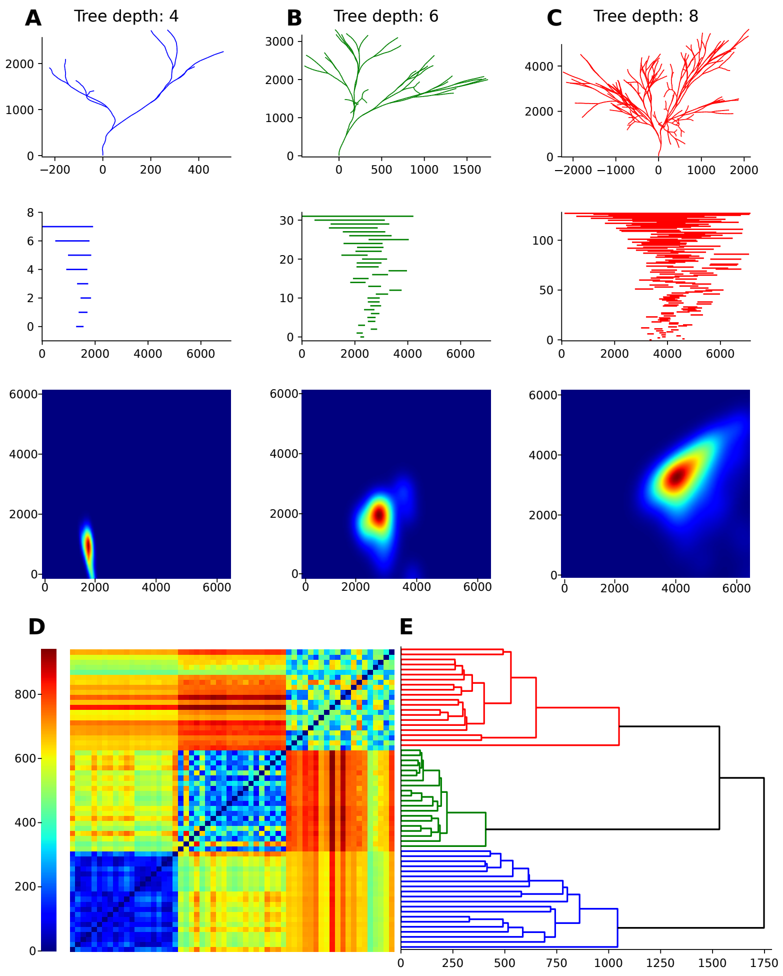

S 7: Groups of trees with different tree depths (4(A), 6(B), 8(C)) can be effectively separated. Larger tree depths result in larger number of branches on the tree ($N_{branches} = 2^{T_d} - 1$). As a result the density of branches increases with the tree depth, and a larger number of topological components is generated in the respective persistence barcodes. The distance matrix (D) indicates the existence of three groups that are identified with high accuracy by a simple dendrogram (E).

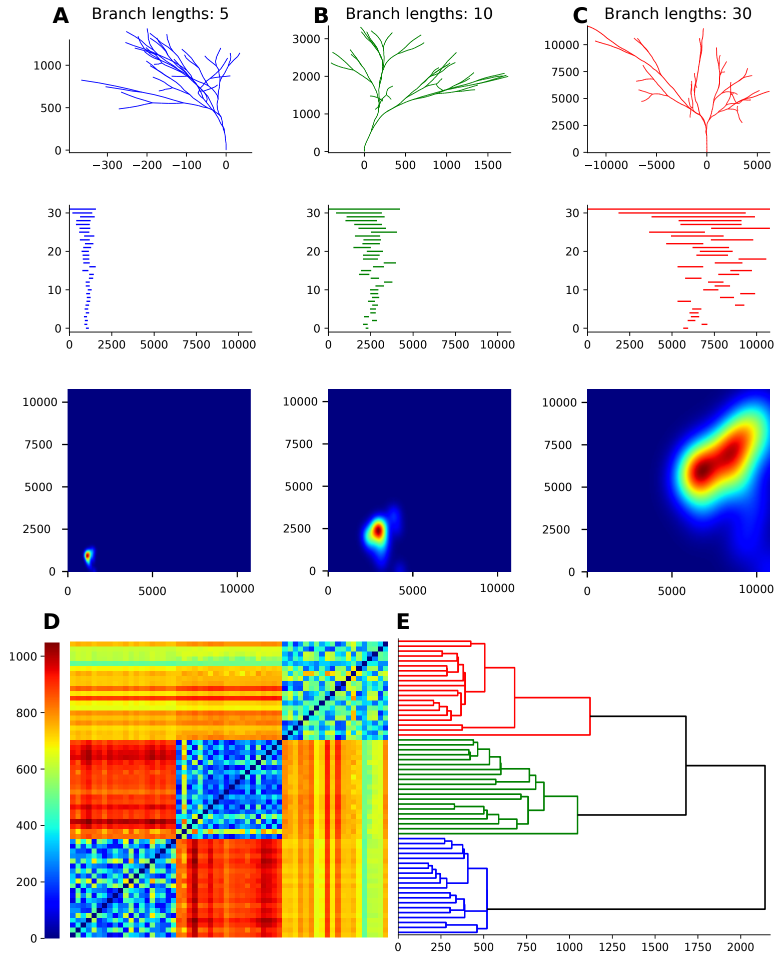

S 8: Groups of trees with different constant branch lengths (5(A), 10(B), 30(C)) can be effectively separated. The length of the branches is reflected in the lengths of the topological components in the respective persistence barcodes. The increasing branch length results in the presence of bars at larger radial distances. The distance matrix (D) indicates the existence of three groups that are identified with high accuracy by a simple dendrogram (E).

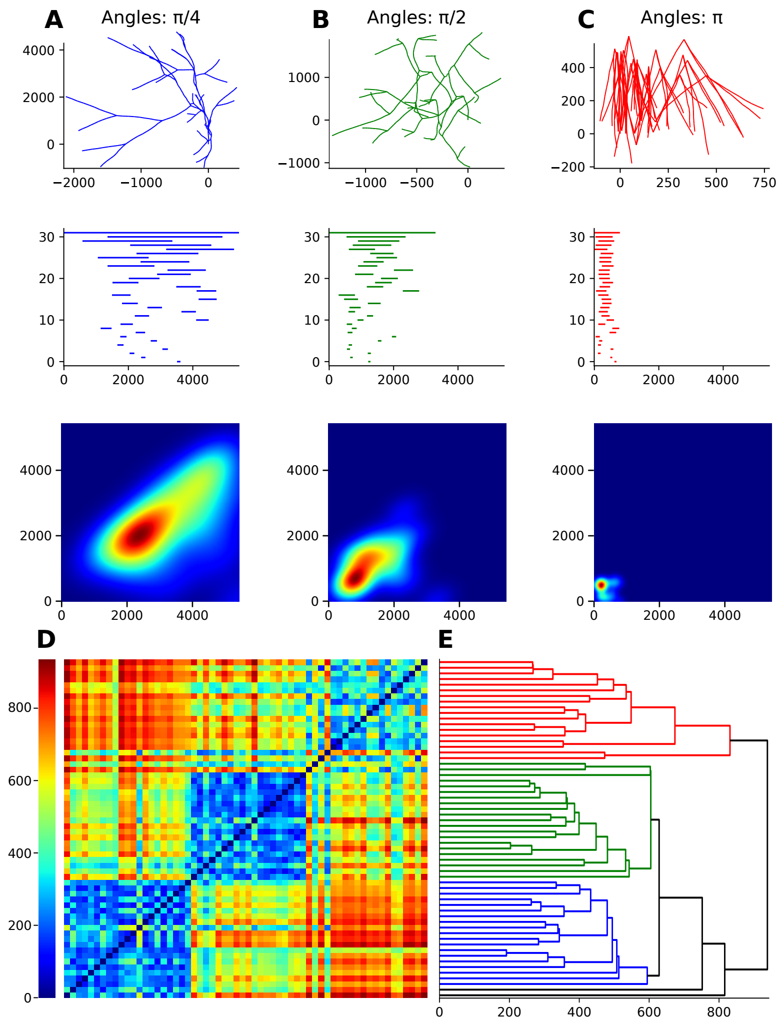

S 9: Groups of trees with different constant branch angles on the $x-y$ plane ($\pi/4$(A), $\pi/2$(B), π(C)) can be effectively separated. The branch angles influence the radial distances of the branches and as a result their respective persistence barcodes. For smaller branch angles the branches of the trees extend to larger radial distances, resulting in longer bars. The distance matrix (D) indicates the existence of three groups that are identified with high accuracy by a simple dendrogram (E). A few mis-classifications are present in the dendrogram (denoted in black). This fact indicates that this distance is not appropriate for 100% accurate separation of branch angles since the branch angles are not directly accounted for in the TMD algorithm. However, the secondary effects of the branch angles can distinguish the trees with very high accuracy (97%).

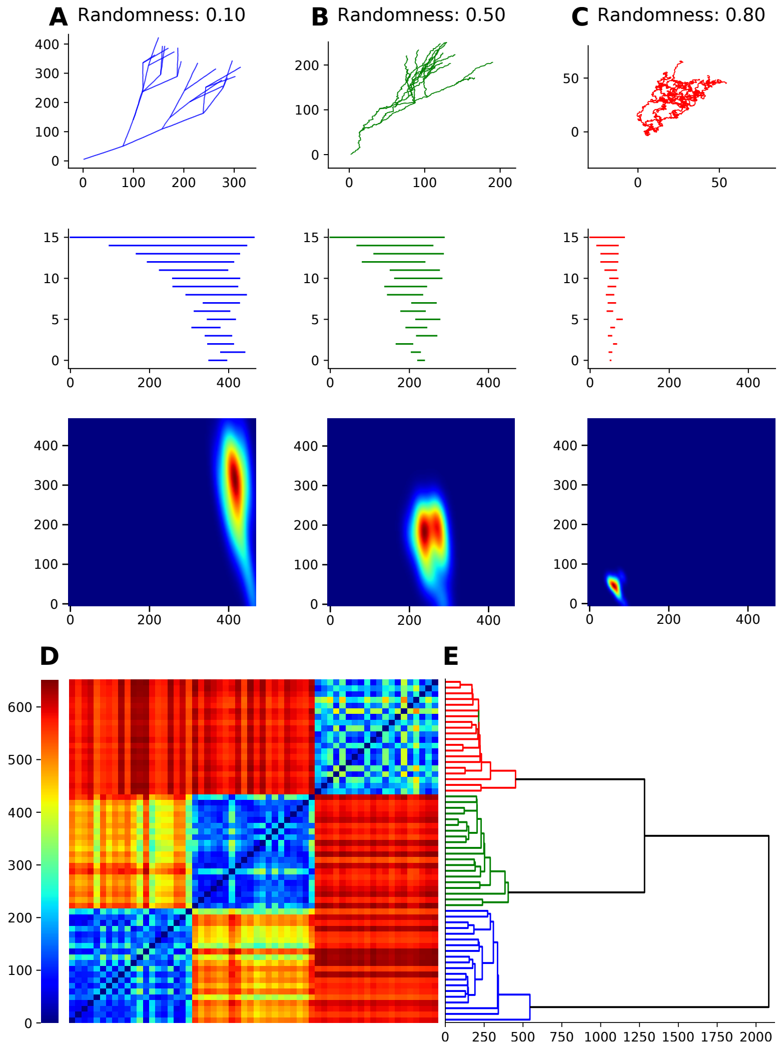

S 10: Groups of trees with different degrees of randomness (0.10(A), 0.50(B), 0.80(C)) can be effectively separated. The degree of randomness influences the extent of individual branches on the trees. For lower values of randomness the trees are less tortuous and extend to larger radial distances. As a result, the trees with smaller degree of randomness generate longer bars in their respective persistence barcodes. The distance matrix (D) indicates the existence of three groups that are identified with high accuracy by a simple dendrogram (E).

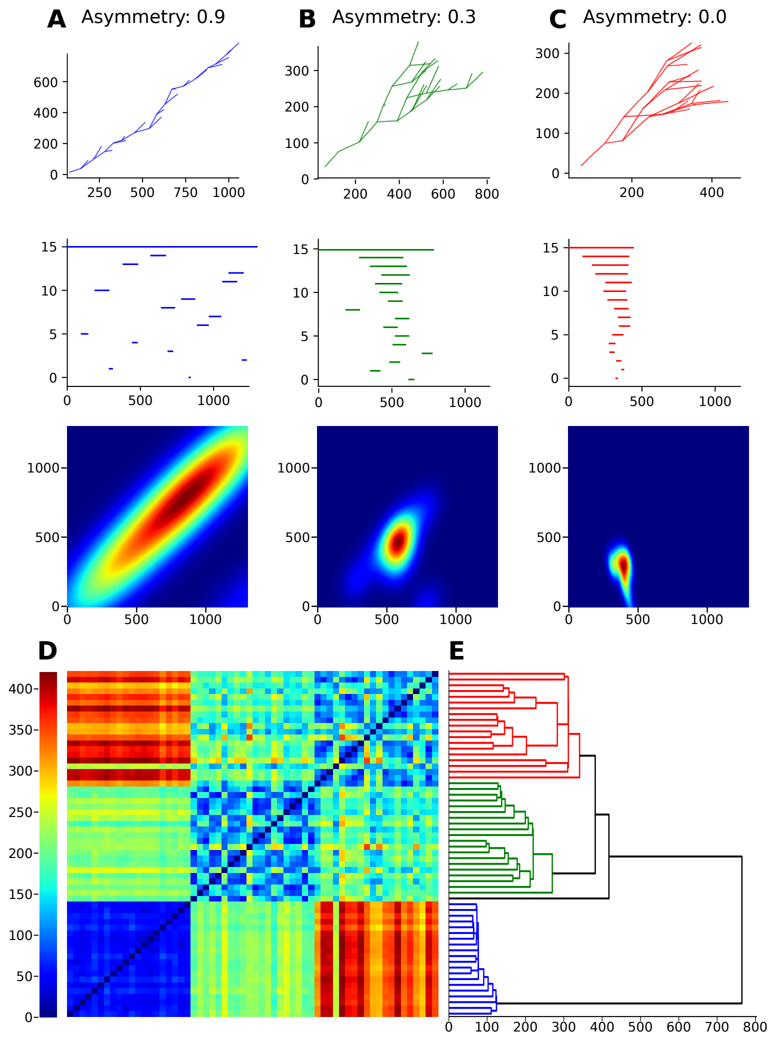

S 11: Groups of trees with different topological patterns that result in different degrees of asymmetry (0.9(A), 0.3(B), 0.0(C)) can be effectively separated. The asymmetry of the branching structure generates distinct patterns in the respective persistence barcodes. Interestingly, the more asymmetric trees (A) result in a more homogeneous distribution of branches in space along the path of the main branch. As a result, the corresponding persistence images are more symmetric around the diagonal. The asymmetry of the trees is reflected in the barcodes by an inverse relation, as the more symmetric trees are encoded in more skewed barcodes. The distance matrix (D) indicates the existence of three groups that are identified with high accuracy by a simple dendrogram (E).

6. Supervised Classification

Supervised classification is a machine learning technique in which a sample dataset (training set) is presented to the algorithm, which then predicts the labels of the individuals that have not been presented (test set).

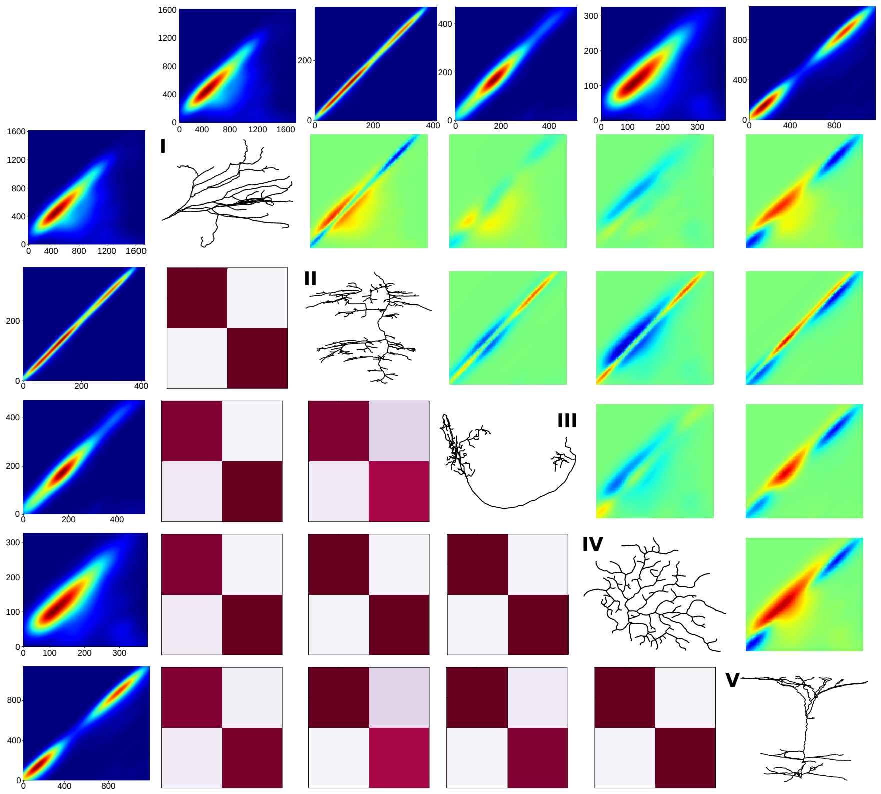

S 12: Results of supervised classification on the dataset of Fig 4 based on the average unweighted persistence images of neuronal morphologies from different species: (I) cat, (II) dragonfly, (III) fruit fly, (IV) mouse and (V) rat. Traditional classification methods measure the degree of separation between two classes, as opposed to the TMD which also reveals the structural principles that differentiate distinct morphological groups. Below the diagonal we illustrate the separation of each pair of groups by presenting the confusion matrices (colorscale from 0 to 1) for the binary classification of the two groups in question. Above the diagonal we present the structural differences between the two groups, as they are revealed by subtracting their unweighted persistence images. Note that since we are studying the structure and not the size differences, the data are not normalized according to the size of the neurons. As a result, the structural differences are unscaled and the relative sizes are presented in the average unweighted persistence images, on the left.

In this section we present the results of the supervised classification that was performed on the trees of the five groups of neurons from different species that are shown in Fig 4. A supervised classification algorithm (Decision Tree)

is trained on the unweighted persistence images. The trained classifier is used to predict the class of trees of the test set. The accuracy of the classifier is defined by the number of the correct predictions divided by the total number of predictions.

The results of the classification are presented with the overall accuracy (percentage) and the confusion matrix. The confusion matrix represents the performance of the classification: true positives are presented in the diagonal, where false positives are presented in non-diagonal elements. A perfect classification would result in ones on the diagonal and zero values everywhere else.

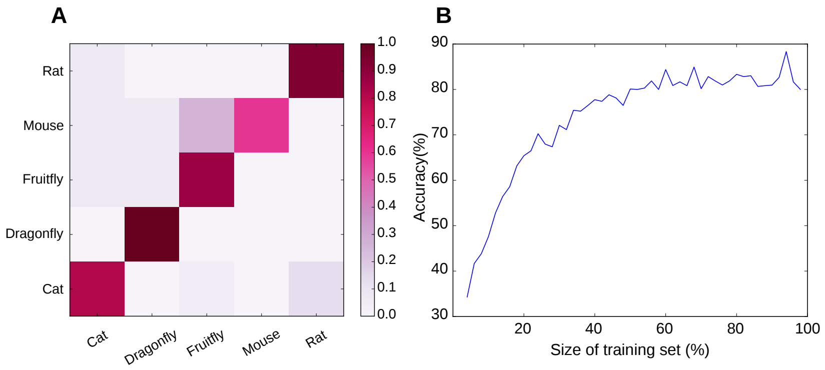

S 13: Supervised classification of neuronal species A. Results of supervised classification, trained on the unweighted persistence images of the five groups of neuronal trees presented in Fig 4. The confusion matrix represents the performance of the classification: true positives are presented in the diagonal, where false positives are presented in non-diagonal elements. Intense red indicates high fraction of data and white shades indicate small fraction of data that correspond to each element of the matrix. The fact that the diagonal is represented in intense red indicates that in most of the cases the classifier accurately predicts the initial group of the neuronal trees. B. For the same dataset (Fig 4) we quantify the accuracy of the supervised classification as the number of correctly predicted labels. The classifier is trained with a subset of the data, as shown in x-axis. As the number of samples that are used for the training is increased the accuracy increases. Note that the accuracy reaches 70% when one fourth of the data (25%, 20 individuals) is used for the training. As a result, a relatively small subset of the data is needed in order to achieve very high accuracy.

6.1 Classification of neuronal trees

The average unweighted persistence images were used for the efficient separation of different morphological classes. The hierarchical clustering (Fig S 12) as well as the supervised classification (Fig S 13A) illustrate the clear separation between the neurons of different species. In addition, by subtracting the persistence images of two groups we can identify the nature of their structural differences, as opposed to traditional methods. Note that the average persistence images have been scaled according to the largest processes for each

species in order to illustrate the scale invariant branching properties of each neuronal type.

For example in Fig S 12, we illustrate the spatial differences of the branching patterns of neuronal trees from the different species of Fig 4. The dragonfly neurons consist of much smaller processes that generate a high concentration of branches around the diagonal, which are not present in other species. Mouse neurons present a wide variety of branch lengths which result in a wider distribution of points around the diagonal compared to all the other species. The rat pyramidal neurons present a tuft at larger radial distances that differentiates them from the other species.

The results of supervised classification, trained on the unweighted persistence images of the five groups of neuronal trees of Fig 4 are shown in Fig S 13A. The higher values in the diagonal of the confusion matrix (true positives) as opposed to small values at the rest of the cases (false positives) indicates that the classifier predicts the actual group of the neuronal trees with high accuracy.

The performance of the classifier as a function of the size of the training set is presented in Fig S 13B for the same dataset (Fig 4). As the number of samples that are used for the training increases the accuracy increases accordingly. Note that the accuracy reaches 70% when one fourth of the data (25%, 20 individuals) is used for the training. As a result, a relatively small subset of the data is needed in order to achieve very high accuracy. The classifier based on the unweighted persistence images is capable to predict the class of neuronal trees even when it is trained with very small datasets. This property is very useful for the classification of neuronal trees, where usually only few data of each class are available.

7. Diversity Index

The diversity index of a community is a quantitative measure that reflects how many different types are present in the dataset and how evenly they are distributed. The diversity increases with the number of types. For a given number of groups, the diversity index is maximized when all groups are equally represented in the dataset. However, most diversity indices behave as if different species had nothing in common.

An alternative method for the characterization of the diversity of a community has been proposed in [11]. The diversity profile, i.e., the graph of the diversity index versus a sensitivity parameter q, describes the shape of the community as the perceived diversity changes with respect to the richness (rare species are influencing the graph for small q) and the dominance of the species (common species almost exclusively define the graph for large q). Therefore, the parameter q represents the inverse of the sensitivity to rare species. The density profile takes into account the *actual similarity* between different groups, as opposed to classical measurements that use the *naive similarity*, i.e., the identity matrix, assuming that different species are completely independent. Based on this method, we generate the diversity profiles of the bi-

ological datasets that have been studied in this paper: neurons of five different species (Fig S 14) and layer five pyramidal cells (Fig S 14).

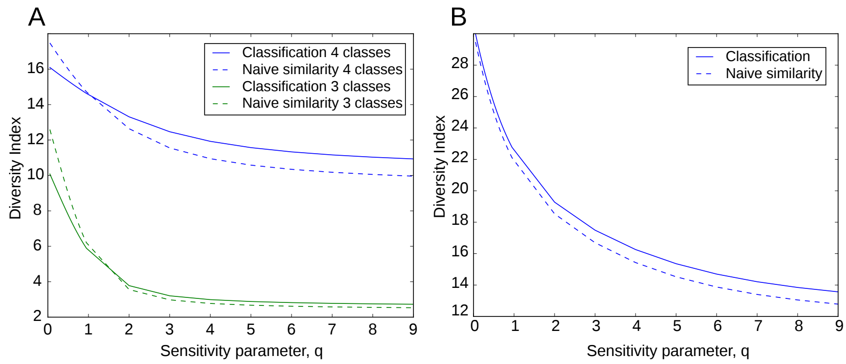

S 14: A. Diversity indices for the species (A) and the L5 pyramidal cells (B).

For the neurons of different species (Fig S 14) the perceived diversity does not change significantly, when we use the actual similarity matrix (solid line) compared to the naive similarity matrix (dashed line). This is due to the fact that neurons of different species are very distinct and therefore their similarity matrix is very close to the identity matrix. It is however interesting to notice that the values of diversity index are much higher in this example compared to the ones of the layer 5 pyramidal cells (Fig S 14), indicating that this dataset is indeed more diverse, as expected from visual examination of the neurons.

On the contrary, the diversity profile of layer 5 pyramidal cells (Fig S 14) is strongly influenced by the similarity matrix in the case of four classes, while this effect is highly reduced in the case of three classes. This indicates that the classification of the neurons in three classes is much more robust. In this case the classes are more distinct and the similarity matrix is closer to the identity matrix.

References

1. DeFelipe J, Lpez-Cruz PL, Benavides-Piccione R, et al. (2013) New insights into the classification and nomenclature of cortical GABAergic interneurons. *Nat Rev Neurosci* 14(3):202–216.
2. Van Pelt J, Verwer RW, Uylings HBM (1989) Centrifugal-order distributions in binary topological trees. *Bull Math Biol.* 51(4):511–536.
3. Van Pelt J, Verwer RW (1983) The exact probabilities of branching patterns under terminal and segmental growth hypotheses. *Bull Math Biol.* 45(2):269–85.
4. Wan Y, Long F, Qu L, Xiao H, Hawrylycz M, Myers EW, Peng H (2015) Blastneuron for automated comparison, retrieval and clustering of 3d neuron morphologies. *Neuroinformatics* 13(4):487–499.
5. Strahler AN, (1952) Hypsometric analysis of erosional topography. *Bull Geol Soc Am* 63:1117–1142.
6. Ledderose J, Sencion L, Salgado H, Arias-Carrion O, Trevino M, (2014) A software tool for the analysis of neuronal morphology data. *Int Arch Med.* 7:6.

7. Sholl DA, (1953) Dendritic organization in the neurons of the visual and motor cortices of the cat. *J Anat.* 87: 387–406.
8. Bille P (2005) A survey on tree edit distance and related problems. *Theoretical Computer Science.*
9. Gillette TA, Ascoli GA (2015) Topological characterization of neuronal arbor morphology via sequence representation: I - motif analysis. *BMC Bioinformatics* 16:216.
10. Gillette T, Hosseini P, Ascoli G (2015) Topological characterization of neuronal arbor morphology via sequence representation: II - global alignment. *BMC Bioinformatics* 16:209.
11. Leinster T, Cobbold C (2012) Measuring diversity: the importance of species similarity. *Ecology* 93(3):477–489.
12. Dey T, Shi D, Wang Y (2015) Comparing graphs via persistence distortion. *SOCG.*
13. Morozov D (2016) *Dionysus, http://mrzv.org/software/dionysus/.*
14. Kerber M, Morozov D, Nigmetov A (2016) *Geometry Helps to Compare Persistence Diagrams.* pp. 103–112.
15. Bubenik P (2015) Statistical topological data analysis using persistence landscapes. *J. Mach. Learn. Res.* 16(1):77–102.
16. Bubenik P, Dłotko P (2015) A persistence landscapes toolbox for topological statistics. *Journal of Symbolic Computations.* abs/1501.00179.
17. Shapira D, Storer JA (2011) Edit Distance with Block Deletions (Algorithms), 4(1) pp. 40–60.
18. Beketayev K, Yeliussizov D, Morozov D, Weber G, Hamann B (2014) *Topological Methods in Data Analysis and Visualization III: Theory, Algorithms, and Applications*, eds. Bremer PT, Hotz I, Pascucci V, Peikert R. (Springer International Publishing, Cham), pp. 151–165.
19. Morozov D, Beketayev K, Weber G (2013) Interleaving distance between merge trees. *Discrete and Computational Geometry* 49:22–45.
20. Dey T, Shi D (2016) *"PersistenceDistortion" software.*
21. Pearson K (1905) The Problem of the Random Walk. *Nature* 72(1865):294.
